# A methodology for RNAi gene knockdown in the marine annelid *Streblospio benedicti* by delivering dsRNA

**DOI:** 10.1242/dev.205644

**Published:** 2026-08-06

**Authors:** Jose Maria Aguilar-Camacho, Christina Zakas

**Affiliations:** Department of Biological Sciences, North Carolina State University, Raleigh, NC 27607, USA

**Keywords:** RNAi, Gene knockdown, dsRNA delivery, Marine polychaetes

## Abstract

RNA interference (RNAi) is a genetic tool that disrupts the expression of selected genes by delivering dsRNA into a specific tissue of an organism, resulting in a gene knockdown. We apply RNAi methods to the marine polychaete S*treblospio benedicti*, which is a model system for studying evolutionary and developmental biology that currently has no established methods for gene expression manipulation. Here, we describe a RNAi gene knockdown methodology using two different approaches depending on developmental stage. We fed bacteria expressing dsRNA to early swimming larvae and microinjected *in vitro* transcribed dsRNA in juveniles. We used two genes for testing: *R-opsin* and *F-actin*. For both developmental stages, gene knockdown was assessed using quantitative real-time PCR. We also visualized RNA expression reduction in larvae using hybridization chain reaction *in situ* hybridization. We show that both delivery methods of larval feeding and juvenile microinjection are sufficient to achieve RNAi gene knockdown, although the specific effect can vary by gene and timing.

## Teaser

**Summary:** Two methodologies for RNAi gene knockdown in the marine annelid *Streblospio benedicti* achieved by delivering dsRNA through feeding and microinjection.

## INTRODUCTION

RNA interference (RNAi) is a well-studied cytological resistance response to double-stranded RNA (dsRNA) that has been widely used for targeted gene expression knock downs. RNAi has been used as a genetic tool to disrupt gene expression, and the RNAi pathway is conserved across many eukaryotes (Mocellin and Provenzano, 2004; Shan, 2010; Lebenzon and Toxopeus, 2024). Due to the efficiency and wide applicability of RNAi, many methods have been developed to introduce dsRNA to model systems. dsRNA can be synthesized *in vitro* and delivered into a specific tissue (i.e. embryos or coelomic cavity) by soaking, injecting, spraying or feeding (Timmons et al., 2001; Kim et al., 2005; Lebenzon and Toxopeus, 2024). However, some taxonomic groups, such as the Lophotrochozoa, have only a few reported successes with these approaches (Feng et al., 2019; Darin et al., 2025).

Feeding dsRNA-expressing bacteria to animals is an effective delivery method in many invertebrate species, including insects, nematodes, planarians and others (Grishok and Mello, 2002; Newmark et al., 2003; Rivera et al., 2011; Iryani et al., 2017; Feng et al., 2019). In aquatic animals, successful gene knockdowns through bacterial feeding have been achieved in the freshwater microcrustacean *Daphnia melanica* (Schumpert et al., 2015), the planarian *Schmidtea mediterranea* (Newmark et al., 2003), the rotifer *Brachionus plicatilis* (Zhang et al., 2024) and the marine worm *Hydroides elegans* (Darin et al., 2025). For filter-feeding invertebrates, successful RNAi through bacterial feeding is limited to the oyster *Crassostrea gigas* (Feng et al., 2019), the marine sponge *Tethya wilhelma*, and the freshwater sponge *Ephydatia* fluviatilis (Rivera et al., 2011). The methods for feeding dsRNA-expressing bacteria in these species vary widely across experiments, but in all cases gene expression was successfully knocked down (Feng et al., 2019).

The marine annelid *Streblospio benedicti* is a model system for studying developmental evolution and evolutionary genetics but, to date, no methods for gene expression manipulation have been established. This annelid has two distinct offspring morphs that differ dramatically in their ontogeny, life-history and morphology (reviewed by Zakas, 2022). The planktotrophic larval morph is an obligately feeding and swimming larva that develops over weeks in the plankton, while the lecithotrophic larval morph is facultatively (non-obligately) feeding with an abbreviated larval phase and notable morphological and behavioral differences (Zakas et al., 2018). As a model for developmental evolution, *S. benedicti* is easily cultured in the lab (McHugh, 2024), although one drawback for establishing functional genetic manipulation tools is that the timing of fertilization cannot be controlled, so typical methods of early embryo microinjections are problematic (Zakas, 2022). For species where microinjections are difficult, or later developmental stages are necessary, delivery of dsRNA through other means becomes more efficient. Here, we present two methods of delivery of dsRNA to knock down gene expression in *S. benedicti.*

We established a gene knockdown by RNAi methodology in *S. benedicti* using two methods depending on developmental stage. We use the bacteria-feeding approach with early swimming feeding larvae and microinjection of *in vitro* transcribed dsRNA in juveniles. To test our approaches, we used two proof-of-principle genes: *R-opsin* (expressed in photoreceptor cells) and *F-actin* (expressed in muscle cells). We selected *R-opsin* because of its known expression pattern near larval eyespots and *F-actin* because it is a ubiquitously expressed gene that is used in other methodology papers. For both developmental stages, we validated RNA knockdown efficiency by qPCR. For larvae, we also visualized reduction in RNA expression using hybridization chain reaction (HCR) *in situ* hybridization (Choi et al., 2018; Aguilar-Camacho et al., 2024). Our study constitutes a successful implementation of gene knockdown by RNAi in a marine annelid species, by feeding dsRNA-expressing bacteria in free-swimming larvae and microinjecting synthesized dsRNA in juveniles.

## RESULTS AND DISCUSSION

### *R-opsin* and *F-actin* expression patterns at different developmental stages

No expression of *R-opsin* is detected at the trochophore stage, even though two pigmented eye spots are present at this developmental stage (see Fig. 1 for development timeline). At the subsequent early swimming stage, *R-opsin* is expressed in six different domains in the larval head. Three of these domains are expressed diagonally on the right side and three on the left side (see Fig. 3). For all later stages, such as swimming larvae, late larvae and early juveniles, there are four pigmented eye spots but six *R-opsin* domains (Fig. 1B-E).

**Fig. 1.**
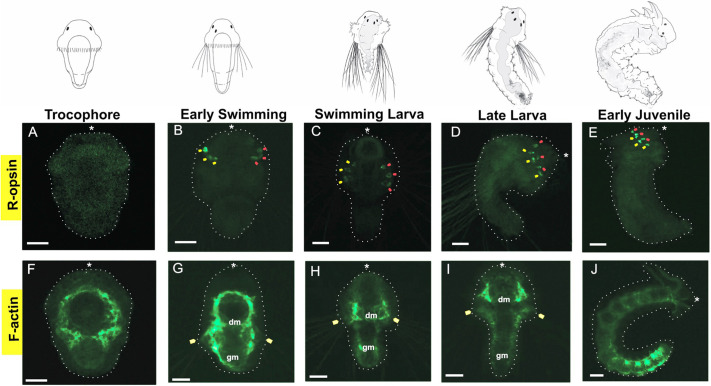
**Wild-type hybridization chain reactions of *R-opsin* and *F-actin* at different developmental stages in the planktotrophic developmental mode of *Streblospio benedicti*.** Drawings for each developmental stage are located at the top of the figure. The numbers of pigmented eyespots (2-4) differs from the number of *R-opsin* domains shown in A-E. Yellow arrowheads in B-E indicate *R-opsin* domains from the right side; red arrowheads in B-E indicate *R-opsin* domains from the left side. White asterisks indicate the anterior end of the larva. Six *R-opsin* domains are present in all the developmental stages except for the trochophore stage; in which no expression was detected. (A) Hybridization chain reaction (HCR) of the *R-opsin* gene at the trochophore stage, ventral view. (B) HCR of the *R-opsin* gene at the early swimming stage (3 eye spots have developed), ventral view. (C) HCR of the *R-opsin* gene at the swimming larval stage (4 eye spots have developed), ventral view. (D) HCR of the *R-opsin* gene at the late larval stage (4 eye spots are still present), lateral view. (E) HCR of the *R-opsin* gene at the early juvenile stage (4 eye spots are still present), lateral view. (F) HCR of the *F-actin* gene at the trochophore stage, ventral view. (G) HCR of the *F-actin* gene at the early swimming larva, ventral view. (H) HCR of the *F-actin* gene at the swimming larval stage, ventral view. (I) HCR of the *F-actin* gene at the late larval stage, ventral view. (J) HCR of the *F-actin* gene at the early juvenile stage, lateral view. Orange arrowheads in G-I indicate chaetal muscles. gm, gastric muscles; dm, dense mass of the ventral head. Scale bars: 20 μm in A,F; 45 μm in B-D,G-I; 50 μm in E,J.

*F-actin* is in muscle fibers throughout larval development*.* At the trochophore stage, a dense irregular mesh formed by muscle fibers is in the anterior end of the larva, and another small irregular-circle mesh is in the posterior end (Fig. 1F). At the early swimming stage, the anterior muscle fibers are reduced and some irregular bands of muscle fibers run dorso-laterally towards the posterior end. Chaetal muscles and gastric muscles are also present (Fig. 1G). At the swimming larval stage, there are some anastomosed longitudinal muscle fibers running dorso-laterally towards the posterior end. A muscle band surrounding the head converges with the longitudinal muscle. Chaetal muscles, gastric muscles and a dense muscle mass in the ventral region of the head are also noticeable (Fig. 1H). At the late larval stage, the expression of *F-actin* is similar to that of the swimming larval stage. However, there are more longitudinal muscle fibers towards the posterior end, as more segments are added (Fig. 1I). Palp muscles, branchia muscles, head muscles and gastric muscles are also noticeable (Fig. 1J). Differences in the expression of *F-actin* at distinct developmental stages have also been reported in other polychaete species (McDougall et al., 2006).

### Larval feeding approach

dsRNA expression for each gene was induced in *E. coli* strain HT115 (DE3) with 0.4 mM IPTG for 4 h at 37°C. Total RNA was isolated from induced bacteria and observed via 1% agarose gel electrophoresis (Fig. S1). A band of dsRNA was visible for bacteria expressing MCS (multiple cloning site) dsRNA, *R-opsin* dsRNA and *F-actin* dsRNA (Fig. S1). Early swimming larvae are obligate feeders and non-selectively consume single-celled algae (McHugh, 2024). Under typical lab conditions, larvae are fed an abundance of *Tetraselmis* sp. algae until they metamorphose into the juvenile stage ∼2 weeks later (McHugh, 2024). We predicted that larvae would also ingest suspended bacteria that are expressing dsRNA. Therefore, we used two treatments: bacteria suspension with and without *Tetraselmis* sp. algae. We sampled two timepoints (4 h and 12 h post-feeding) to test for gene expression reduction.

We fed early swimming larvae (3-eye stage larvae that are ∼5 days old) bacteria expressing dsRNA for 3 h in two separate experiments. As a control, larvae were fed equal amounts of bacteria containing a plasmid expressing only MCS dsRNA with no gene sequence (Fig. S1). This controls for the potential side effects of introducing dsRNA without targeting a specific gene. The MCS of the L4440 plasmid (340 bp) has been used in other species of invertebrates as samples of non-targeting dsRNA (Ghosh and Gundersen-Rindal., 2017; De-Souza et al., 2019). As early-swimming larvae are capable of feeding, but have not yet developed a fourth eyespot, this was an ideal stage to test dsRNA knockdown efficiency using *R-opsin*.

### Larval feeding results

We quantified gene expression reduction in our treatments using qPCR and visualized mRNA expression patterns using HCR *in situ* for larval feeding experiments. Early swimming larvae of the feeding experiment showed a reduction of *R-opsin* expression, compared to controls of targeting non-dsRNA (MCS dsRNA) and non-treatment control samples (ANOVA, Tukey's post-hoc, *P*<0.05) in all four cases tested (Fig. 2; Table S1). Therefore, 3 h of ingesting dsRNA-expressing bacteria in the early-swimming larvae was sufficient time to reduce gene expression for the *R-opsin* gene (Fig. 2; Table S1). However, no significant differences were detected for *F-actin* (ANOVA, Tukey's post-hoc, *P*<0.05; (Fig. S2; Table S2). Using HCR *in situ*, we visualized the change in RNA expression in the early swimming larvae (Fig. 1B). Larvae that ate bacteria expressing *R-opsin* dsRNA at the two timepoints (with and without algae) showed a reduction in *R-opsin* domains. Only 4 or 5 visible *R-opsin* domains were present compared to the larvae samples that fed MCS dsRNA, which have 6 (Fig. 3B,D,F,H; Table S3; Figs S3 and S4). Interestingly, the reduction of these domains is on the left side of the larval head, which is the side that forms the fourth eye later in development. There is also a noticeable reduction in brightness in the most-posterior *R-opsin* spot that does not overlap with an eyespot (Fig. 3F,H). This indicates that the reduction of *R-opsin* domains is not uniform across the larvae, but rather the reduction is restricted to the side of the newly forming eye spots. We do not know why there is a patchy reduction in expression rather than a ubiquitous reduction, but it could indicate that the mRNA has a long half-life and persists where established, while newly transcribed RNAs may be more susceptible to the RNAi targeting. No significant differences were detected for the *F-actin* larval feeding experiment using qPCR and HCR *in situ* (although we do see a difference in our juvenile injection results; Figs S2, S5). Similar approaches in sponge and cnidarian species showed a reduction of actin expression using qPCR, but no noticeable phenotypic differences based on phalloidin staining (Rivera et al., 2011; Kyslik et al., 2024). In contrast, the sea anemone *Aiptasia pallida* showed altered tissue morphology with actin reduction when high doses and long incubation time were used (Dunn et al., 2007). It is possible that a visual phenotype could be achieved in *S. benedicti* with longer exposure or higher dosage of dsRNA than was tested in this feeding experiment.

**Fig. 2.**
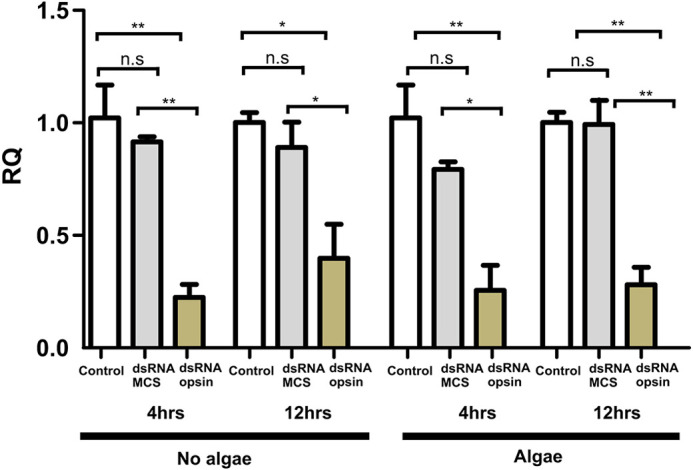
**Larval feeding of R-opsin dsRNA.** Quantitative PCR (qPCR) analysis of the *R-opsin* expression levels with and without algae at two timepoints post feeding. *Y*-axis denotes relative levels of expression (RQ). Data are mean±s.e.m. of *n*=3 replicates. One-way analysis of variance with Tukey's multiple comparison test for each treatment were calculated and statistical significances in all cases were defined as: n.s., no significant differences; *P<0.05, **P<0.001. MCS, multiple cloning site of the L4440 plasmid.

**Fig. 3.**
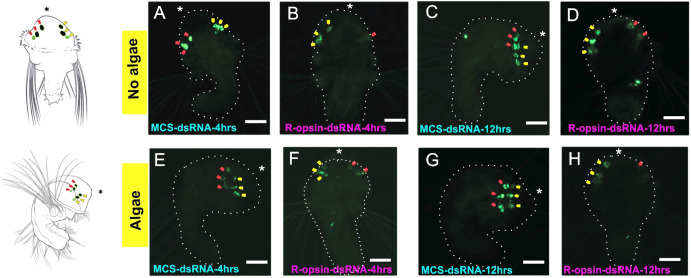
**Hybridization chain reaction *in situ* hybridization of the *S. benedicti R-opsin* dsRNA and MCS dsRNA larval feeding experiment.** The drawings show the swimming larval stage with four eyespots (black) overlapping four *R-opsin* domains (green) and two additional *R-opsin* domains in the larval head. Asterisks indicate the anterior end of the larva. Top left drawing is dorsal view. Bottom left drawing is lateral view. Yellow arrowheads indicate *R-opsin* domains from the right side and red arrowheads indicate *R-opsin* domains from the left side. White dashed lines outline the body for each individual sample. Scale bars: 45 μm. (A) Control early swimming larva from the feeding experiment MCS dsRNA/no algae/4 h experiment. Dorsal view shows all six *R-opsin* domains. (B) Early swimming larva from the *R-opsin* dsRNA/no algae/4 h experiment. Ventral view shows four *R-opsin* domains – three on the right and one on the left. (C) Control early swimming larva from the MCS dsRNA/no algae/12 h experiment. Lateral view shows all six *R-opsin* domains. (D) Early swimming larva from the *R-opsin* dsRNA/no algae/12 h experiment ventral view shows five *R-opsin* domains – three on the right and two on the left. (E) Control early swimming larva from the MCS dsRNA/algae/4 h experiment. Lateral view shows six *R-opsin* domains. (F) Early swimming larva from the *R-opsin* dsRNA/algae/4 h experiment. Ventral view shows five R-opsin domains – three on the right and two on the left. (G) Control early swimming larva from the MCS dsRNA/algae/12 h experiment. Lateral view shows six *R-opsin* domains. (H) Early swimming larva from the *R-opsin* dsRNA/algae/12 h experiment. Ventral view shows four *R-opsin* domains: three on the right and one on the left. The total number of observations and supporting phenotypes for each treatment and timepoint in Table S3.

The different results between the two genes tested here could indicate that ubiquitously expressed or critical genes – which may have genetic redundancy – are less susceptible to knockdown by dsRNA using these conditions. However, variability in the efficiency of dsRNA knockdowns between genes within a species is a common phenomenon (Kamath et al., 2000; Lebenzon and Toxopeus, 2024). Small amounts of dsRNA are effective in *C. elegans* to produce a gene knockdown (Kamath et al., 2000). It is possible that higher doses of dsRNA could potentially increase the reduction of the expression levels of a particular gene in *S. benedicti.* In addition, there is one gene copy of *DICER* and one gene copy for *SID-1* in the genome of *S. benedicti* (Table S6). SID-1 acts as a passive importer, allowing dsRNA to move between cells in *C. elegans* (Ravikumar et al., 2019). The role of *SID-1* in cellular propagation is variable across invertebrates (Wang and Hunter, 2017) and its role in *S. benedicti* has not been verified yet.

### Juvenile microinjection approach

We injected *in vitro* transcribed dsRNA (120 ng/μl) of *R-opsin* and *F-actin* into 3-week-old juveniles (annelids; Chen et al., 2020). MCS dsRNA (120 ng/μl) was used for the control samples targeting non-dsRNA for side effects of dsRNA and the non-treatment control samples were injected with rhodamine (see Materials and Methods).

We tested gene expression differences using qPCR at two timepoints (6 h and 12 h after microinjection). At both timepoints the *R-opsin*-injected animals had reduced expression levels (ANOVA, Tukey's post-hoc, *P*<0.05), compared to the control samples (Fig. 4A; Table S4). Animals injected with *F-actin* dsRNA also had reduced expression (ANOVA, Tukey's post hoc, *P*<0.05) but only at one time point: 12 h after injection (Fig. 4B; Table S5).

**Fig. 4.**
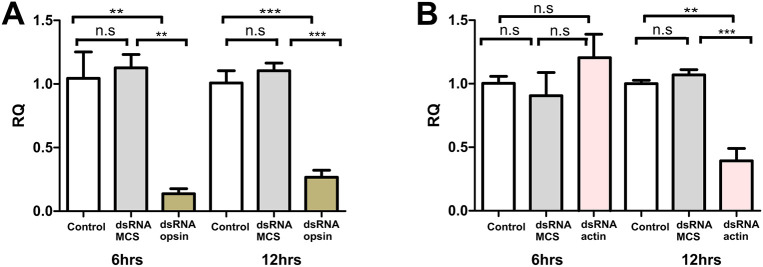
**Quantitative PCR expression levels for microinjected juveniles.** (A) Quantitative PCR (qPCR) analysis of the *R-opsin* expression levels. (B) qPCR analysis of the *F-actin* expression levels. *Y*-axis denotes relative levels of expression (RQ). Data are mean±s.e.m. of *n*=3 replicates. One-way analysis of variance with Tukey's multiple comparison test for each treatment were calculated and statistical significances in all cases were defined as: n.s., no significant differences; **P<0.001, ***P<0.0001. MCS, multiple cloning site of the L4440 plasmid.

In summary, *R-opsin* expression was reduced at two timepoints after microinjection of dsRNA, and *F-actin* was reduced at only the second timepoint (Fig. 4A,B; Tables S4,S5). These results demonstrate that there is variability in the knockdown efficiency and timing between genes, but the delivery method by microinjection is sufficient to achieve expression reduction in the target gene. These results illustrate that maximizing knockdown efficiency requires troubleshooting the timing, delivery method and dsRNA sequence for individual target genes as there is variability; however, both the approaches shown here can work. This variability in knockdown efficiency is expected: it has been demonstrated that in some arthropods, knockdown efficiency of opsin and actin genes depends on the dsRNA concentration and time after injection (Fan et al., 2022; Huang et al., 2023; Abbà et al., 2019; Rosa et al., 2012). While incubation time and concentration can be maximized, both microinjection and feeding approaches for RNAi can achieve gene expression knockdown in *S. benedicti.*

### Conclusions

We have demonstrated that both larval feeding and juvenile microinjection are sufficient delivery mechanisms for dsRNA to result in RNAi in target genes. This is a major step for gene expression manipulation experiments in post-embryonic developmental stages in marine species. The efficiency of gene knockdown can vary depending on the gene, timing or dosage of dsRNA, but this is typical for RNAi-based approaches. We can conclude that an appropriate delivery method depends on the target gene and the developmental stage but dsRNA for RNAi knockdown is achievable in *S. benedicti*.

## MATERIALS AND METHODS

### Animal culturing

Planktotrophic larvae were obtained from brooding females from maintained laboratory cultures (see McHugh, 2024).

### Feeding early swimming larvae with bacteria expressing dsRNA

*R-opsin* (1224 bp) and *F-actin* (1131 bp) genes were identified in the transcriptome of *S. benedicti* as shown by Aguilar-Camacho et al. (2024) (Table S6). Fragments of *R-opsin* (467 bp) and *F-actin* (584 bp) were synthesized (Genscript) and inserted into the L4440 (Adgene) plasmid vector (SacI-HindIII). This vector contains two convergent T7 polymerase promoters in opposite orientation separated by a multicloning site that is used for the induction of dsRNA in *E. coli* cells (Timmons et al., 2001). HT115 (DE3) competent cells were prepared using standard CaCl_2_ methodology and were transformed with the plasmid vectors by heat shock. Transformed cells were grown overnight onto LB agar plate with ampicillin (100 μg/ml) at 37°C. A positive bacteria colony for each gene was chosen for an overnight culture in a 5 ml LB medium with ampicillin (100 μg/ml) and tetracycline (50 μg/ml) at 37°C. The next day, the overnight bacteria culture was inoculated (500 μl) into a 50 ml LB medium with ampicillin (100 μg/ml) and tetracycline (50 μg/ml) at 37°C until OD_595_=0.4 was reached. Expression of dsRNA was induced with 0.4 mM IPTG for 4 h at 37°C under agitation. Induced bacteria cultures were centrifuged (845 ***g***×3 min) and the pellet washed twice with artificial sea water.

For larval feeding, ∼1×10^8^ cells/ml of *E. coli* were added to early swimming larvae with or without *Tetraselmis* sp. algae (∼2000 cells/ml; Carolina Biological Supply) for 3 h in 1 well of a 6-well plate (early swimming larvae of *S. benedicti* are fed *Tetraselmis* sp. algae in lab culture). Bacteria that expressed either MCS dsRNA or *R-opsin* dsRNA were fed to planktotrophic larvae. Experiments longer than 3 h resulted in a dense layer at the bottom of the well that may have contributed to some larval mortality. Therefore, we limited the feeding duration to 3 h.

Clutches of planktotrophic larvae (>300 individuals) from the same brood were divided into treatments and control samples of 25 individuals per well. We used three replicates for each timepoint and treatment, and the experiment was repeated twice. Samples targeting non-dsRNA were fed bacteria from a transformed positive colony with the L4440 plasmid where no gene was inserted (MCS dsRNA) and the non-treatment control samples were wild-type early swimming larvae (three replicates of 25 larvae). After feeding planktotrophic larvae for 3 h, the larvae were washed twice with artificial seawater. Gene expression was measured at two timepoints, 4 h and 12 h, after washing the larvae samples that were fed. Larvae from each treatment were either collected for RNA extraction (for qPCR) or fixed for HCR imaging consisting of three replicates (Fig. S6).

### Microinjection of *in vitro* synthesized dsRNA into juveniles

*R-opsin* (467 bp) and *F-actin* (602 bp) fragments were amplified by PCR from cDNA of a 3-week-old planktotrophic juvenile using the following primers: 5′-TAATACGACTCACTATAGGGCCAGTACCAAGCACCTTCGGAC (forward for *R-opsin*), 5′-TAATACGACTCACTATAGGGGAAGAACATAGGCACTCCAAAC (reverse for *R-opsin*), 5′-TAATACGACTCACTATAGGGGACGATGATGTTGCTGCTCTCG (forward for *F-actin*) and 5′-TAATACGACTCACTATAGGGCGCTCGGTAAGGATCTTCATCA (reverse for *F-actin*). For controls, a purified L4440 plasmid was used as a template to amplify the MCS using the following primers: 5′-TTGGGTAACGCCAGGGTTTT (Backbone forward) and 5′-TACCGCCTTTGAGTGAGCTG (Backbone reverse) (Table S8). PCR products for each gene were purified by using the DNA Clean & Concentrator kit (Zymo Research) and used as template for dsRNA *in vitro* transcription using the HiScribe T7 High Yield RNA Synthesis Kit (NEB). The RNA pellet for each gene was resuspended in 50 μl of Molecular Biology Water (Sigma) and stored at −80°C until further use. We chose 3-week-old lecithotrophic juveniles for injection as other developmental stages, such as early juveniles (earlier than two weeks) and swimming lecithotrophic larvae, leaked the solution after microinjection. In contrast, 2-month-old juveniles and adults have a dense epidermal cuticle that is difficult to penetrate. Prior to injection, 3-week-old juveniles were relaxed by using 700 mM of MgCl_2_ (Sigma) with artificial sea water for 30 min at room temperature. Each worm was then placed into a small petri dish with artificial sea water in which half of the petri dish was filled with 3% molecular biology grade agarose (Apex). This agarose structure was used as a ‘wall’ and helped throughout the injection of the worms. Microinjection was applied with an InjectMan 4 Micromanipulation Workstation (Eppendorf) connected to a Femtojet 4i system (Eppendorf) that was observed using an AXIO Observer A1 Inverted Fluorescence Microscope (Zeiss). Heat-pulled quartz needles (QF100-70-10; Sutter Instruments) were pulled using a micropipette puller (P-2000; Sutter Instruments). They were backfilled with 120 ng/μl dsRNA of the target gene (either *R-opsin* or *F-actin*) and 1.5% (w/v) red rhodamine-dextran (70,000 MW, Neutral, Invitrogen). In a preliminary experiment, we injected two different *R-opsin* dsRNA concentrations, 120 ng/μl and 300 ng/μl, into juvenile worms. However, we decided to use the lowest concentration because there was a reduction of the expression levels of the *R-opsin* gene in samples injected with the lowest concentration after 6 h. We considered that 120 ng/μl of dsRNA was sufficient for our experiment.

Samples targeting non-dsRNA were injected with MCS dsRNA (120 ng/μl dsRNA) and rhodamine-dextran. The solution was injected as above (Fig. S7A,B). We injected (injection pressure 150, injection time 1.20, compensation pressure 60) both the gut lumen and the body cavity to maximize dsRNA introduction. We tested gene expression differences at two timepoints (6 h and 12 h after injection) with three biological replicates for no treatment control samples (juveniles injected only with rhodamine-dextan), samples targeting non-dsRNA and experiment samples for each gene tested. After injection, individuals were transferred into a small petri dish with artificial seawater and stored in a small incubator at 20°C until they were used for total RNA extraction. A single individual worm was considered as one replicate and three replicates for each timepoint were performed.

### Total RNA extraction and qPCR analyses

For all experiments, total RNA was extracted using the Monarch Total RNA prep kit (NEB). To test for dsRNA expression, induced bacteria (<5×10^7^) were centrifuged at 845 ***g***×3 min in an Eppendorf tube and a portion of the supernatant removed for RNA extraction. Each replicate (25 larvae) of the feeding experiment and the injected juvenile worms were transferred into an Eppendorf tube and centrifuged at 376 ***g***×3 min. Artificial seawater was removed prior to RNA extraction. RNA was extracted following the Monarch (NEB) protocol. RNA was stored at −80°C until further use. Total RNA from bacteria expressing dsRNA was loaded into a 1% molecular biology grade agarose gel (Apex) for 45 min at 80 V and the gel imaged using a Accuris Instruments SmartDoc 2.0 (Nova Tech). Reverse transcription (RT) was carried out using the Monarch Protoscript II First Strand cDNA synthesis Kit (NEB). RNA quantifications were determined by using the Qubit 4 Fluorometer (Thermo Fisher) (Table S7). Primers for *R-opsin* are: 5′-CCTCACGGTCAACAGCATCT (forward) and 5′-CTGCCTTCATGGGGTTGGAT (reverse), 158 bp product length (primer efficiency: 92.784). Primers for *F-actin* are: 5′-TGGTGGGTATGGGCCAGAAG (forward) and 5′-CCAACTGGGACGACATGGAGA (reverse), 120 bp product length (primer efficiency: 94.682). Three independent repeats (3× qPCR) for each of the replicates were examined and the samples were subjected to 40 cycles of amplification in a QuantStudio 6 Pro Real-Time PCR System (Thermo Fisher) and the Kapa SYBR Fast qPCR master mix was used (Roche). All relative levels of gene expression of the *R-opsin*, *F-actin* and control samples of non-targeting dsRNA were normalized to the housekeeping gene *Elongation Factor 1* (*EF-1*) with the non-treatment control samples for each independent experiment (Ct values of *EF-1* were stable in all the samples tested). Primers of *EF-1* are: 5′-TGTGGAGACTTTCAGCGAGT (forward) and 5′-CGAGGCTCACTTCTTCTTGC (reverse), 163 bp product length (primer efficiency: 94.238) (Table S8). Data were analyzed using the ΔΔCt method and the relative levels of expression were calculated by the 2^−ΔΔCt^ method, which is expressed as RQ (relative quantity) (Rao et al., 2013; Livak and Schmittgen, 2001; Aguilar-Camacho and McCormack, 2019).

### Statistical analyses

RQ data values (2^−ΔΔCt^ or 2^−ΔΔCt−1^) were compared between the samples of the experiment with their respective RQ data values of the control samples. One-way analysis of variance with Tukey's multiple comparison test for each treatment were calculated and statistical significances in all cases were defined at *P*<0.05. These analyses were performed with the GraphPad Prism software (Tables S1, S2, S4 and S5).

### Hybridization chain reaction (HCR) *in situ* hybridization and imaging

Multiplex probes of *R-opsin* and *F-actin* transcripts of *S. benedicti* were designed using the HCR3.0 Probe Maker (Kuehn et al., 2022; Tables S9 and S10; 6) (20 bp from 5′ end of the sense RNA before starting to hybridize, 4 bp max length for polyA and polyT, and 8 bp max length for polyC and polyT). Sequences were synthesized into DNA oligo pools (50 pmol DNA Pools Oligo Pool) from Integrated DNA Technologies and resuspended to 1 pmol/μl in nuclease free water (NEB). Animals were fixed in 4% paraformaldehyde at 4°C overnight, transferred stepwise into 100% methanol and kept at−20°C. Samples were rehydrated through a methanol/DEPC-treated PBSt series. HCR was performed as previously described (Aguilar-Camacho et al., 2024). HCR samples were mounted in Slowfade Glass (Thermo Fisher) with DAPI and kept at 4°C until imaging and imaged using Zeiss Laser Scanning Confocal Microscope LSM 880. *Z*-stack images (40 layers) were processed in ImageJ (Sheffield, 2007). We imaged 2-5 individuals from each sample of each developmental stage of the two genes (*R-opsin* and *F-actin*). For the *R-opsin* feeding experiment, each treatment and timepoint had twenty larvae analyzed, and the *R-opsin* domains in each sample were counted (See Table S3).

## Supplementary Material



10.1242/develop.205644_sup1 Supplementary information

Table S1. Ct values of the feeding experiment (*R-opsin*) and the ANOVA test for each treatment.

Table S2. Ct values of the feeding experiment (*F-actin*) and the ANOVA test for each treatment.

Table S4. Ct values of the microinjection experiment (*R-opsin*) and the ANOVA test for each treatment.

Table S5. Ct values of the microinjection experiment (*F-actin*) and the ANOVA test for each treatment.

Table S6. DNA sequences of the *R-opsin* and *F-actin* (from the transcriptome and the gene inserted into the plasmid).

Table S7. RNA concentration of the larvae from the larvae from the feeding experiment and injected juveniles from the dsRNA microinjections.

Table S9. HCR hairpin probes of the *F-actin* gene.

Table S10. HCR hairpin probes of the *R-opsin* gene.

## References

[DEV205644C1] Abbà, S., Galetto, L., Ripamonti, M., Rossi, M. and Marzachì, C. (2019). RNA interference of muscle actin and ATP synthase beta increases mortality of the phytoplasma vector *Euscelidius variegatus*. *Pest Manag. Sci.* 75, 1425-1434. 10.1002/ps.5263 30417535

[DEV205644C2] Aguilar-Camacho, J. M. and McCormack, G. P. (2019). Silicatein expression in Haliclona indistincta (Phylum Porifera, Order Haplosclerida) at different developmental stages. *Dev. Genes Evol.* 229, 35-41. 10.1007/s00427-019-00627-7 30756180

[DEV205644C3] Aguilar-Camacho, J. M., Harry, N. D. and Zakas, C. (2024). Comparative Hox genes expression within the dimorphic annelid *Streblospio benedicti* reveals patterning variation during development. *EvoDevo* 15, 12. 10.1186/s13227-024-00231-5 39334480 PMC11438215

[DEV205644C5] Chen, C. P., Fok, S. K. W., Hsieh, Y. W., Chen, C. Y., Hsu, F. M., Chang, Y. H. and Chen, J. H. (2020). General characterization of regeneration in *Aeolosoma viride* (Annelida, Aeolosomatidae). *Invertebr. Biol.* 139, e12277. 10.1111/ivb.12277

[DEV205644C6] Choi, H. M., Schwarzkopf, M., Fornace, M. E., Acharya, A., Artavanis, G., Stegmaier, J., Cunha, A and Pierce, N. A. (2018). Third-generation in situ hybridization chain reaction: multiplexed, quantitative, sensitive, versatile, robust. *Development*, 145, dev165753. 10.1242/dev.165753 29945988 PMC6031405

[DEV205644C7] Darin, E., Farrell, M. V., Ali, T. N., Rivera Alfaro, J., Malter, K. E. and Shikuma, N. J. (2025). MyD88 knockdown by RNAi prevents bacterial stimulation of tubeworm metamorphosis. *Proc. Natl Acad. Sci. USA* 122, e2505805122. 10.1073/pnas.2505805122 40455987 PMC12167997

[DEV205644C8] De-Souza, E. A., Camara, H., Salgueiro, W. G., Moro, R. P., Knittel, T. L., Tonon, G., Pinto, S., Pinca, A. F. P., Antebi, A., Pasquinelli,A. E. et al. (2019). RNA interference may result in unexpected phenotypes in *Caenorhabditis elegans*. *Nucleic Acids Res.*, 47, 3957-3969. 10.1093/nar/gkz154 30838421 PMC6486631

[DEV205644C9] Dunn, S. R., Phillips, W. S., Green, D. R. and Weis, V. M. (2007). Knockdown of actin and caspase gene expression by RNA interference in the symbiotic anemone *Aiptasia pallida*. *Biol. Bull.* 212, 250-258. 10.2307/25066607 17565114

[DEV205644C10] Fan, Z., Zhang, Z., Zhang, X., Kong, X., Liu, F. and Zhang, S. (2022). Five visual and olfactory target genes for RNAi in *Agrilus planipennis*. *Front. Genet.* 13, 835324. 10.3389/fgene.2022.835324 35186047 PMC8855093

[DEV205644C11] Feng, D., Li, Q. and Yu, H. (2019). RNA interference by ingested dsRNA-expressing bacteria to study shell biosynthesis and pigmentation in *Crassostrea gigas*. *Mar. Biotechnol.* 21, 526-536. 10.1007/s10126-019-09900-2 31093810

[DEV205644C12] Ghosh, S. K. B. and Gundersen-Rindal, D. E. (2017). Double strand RNA-mediated RNA interference through feeding in larval gypsy moth, *Lymantria dispar* (Lepidoptera: Erebidae). *Eur. J. Entomol.* 114, 170-178. 10.14411/eje.2017.022

[DEV205644C13] Grishok, A. and Mello, C. C. (2002). RNAi (nematodes: *Caenorhabditis elegans*). *Adv. Genet.* 46, 339-360. 10.1016/S0065-2660(02)46012-9 11931230

[DEV205644C14] Huang, M., Meng, J. Y., Zhou, L., Yu, C. and Zhang, C. Y. (2023). Expression and function of opsin genes associated with phototaxis in *Zeugodacus cucurbitae* Coquillett (Diptera: Tephritidae). *Pest Manag. Sci.* 79, 4490-4500. 10.1002/ps.7651 37418556

[DEV205644C15] Iryani, M. T. M., MacRae, T. H., Panchakshari, S., Tan, J., Bossier, P., Wahid, M. E. A. and Sung, Y. Y. (2017). Knockdown of heat shock protein 70 (Hsp70) by RNAi reduces the tolerance of *Artemia franciscana* nauplii to heat and bacterial infection. *J. Exp. Mar. Biol. Ecol.* 487, 106-112. 10.1016/j.jembe.2016.12.004

[DEV205644C16] Kamath, R. S., Martinez-Campos, M., Zipperlen, Fraser, A. G. and Ahringer, J. (2000). Effectiveness of specific RNA-mediated interference through ingested double-stranded RNA in *Caenorhabditis elegans*. *Genome Biol.* 2, 2000. 10.1186/gb-2000-2-1-research0002 PMC17598 11178279

[DEV205644C17] Kim, D.-H., Behlke, M. A., Rose, S. D., Chang, M.-S., Choi, S. and Rossi, J. J. (2005). Synthetic dsRNA Dicer substrates enhance RNAi potency and efficacy. *Nat. Biotechnol.* 23, 222-226. 10.1038/nbt1051 15619617

[DEV205644C18] Kuehn, E., Clausen, D. S., Null, R. W., Metzger, B. M., Willis, A. D. and Özpolat, B. D. (2022). Segment number threshold determines juvenile onset of germline cluster expansion in *Platynereis dumerilii*. *J. Exp. Zool. B Mol. Dev. Evol.* 338, 225-240. 10.1002/jez.b.23100 34793615 PMC9114164

[DEV205644C19] Kyslík, J., Born-Torrijos, A., Holzer, A. S. and Kosakyan, A. (2024). RNAi-directed knockdown in the cnidarian fish blood parasite *Sphaerospora molnari*. *Sci. Rep.* 14, 3545. 10.1038/s41598-024-54171-0 38347054 PMC10861503

[DEV205644C20] Lebenzon, J. E. and Toxopeus, J. (2024). Knock down to level up: reframing RNAi for invertebrate ecophysiology. *Comp. Biochem. Physiol. A Mol. Integr. Physiol.* 297, 111703. 10.1016/j.cbpa.2024.111703 39029617

[DEV205644C21] Livak, K. J. and Schmittgen, T. D. (2001). Analysis of relative gene expression data using real-time quantitative PCR and the 2− ΔΔCT method. *Methods* 25, 402-408. 10.1006/meth.2001.1262 11846609

[DEV205644C22] McDougall, C., Chen, W. C., Shimeld, S. M. and Ferrier, D. E. (2006). The development of the larval nervous system, musculature and ciliary bands of *Pomatoceros lamarckii* (Annelida): heterochrony in polychaetes. *Front. Zool.* 3, 16. 10.1186/1742-9994-3-16 17032451 PMC1615870

[DEV205644C23] McHugh, K. (2024). Animal Care Protocol: Streblospio benedicti. *protocols.io* 10.17504/protocols.io.eq2lyw24wvx9/v1

[DEV205644C24] Mocellin, S. and Provenzano, M. (2004). RNA interference: learning gene knock-down from cell physiology. *J. Transl. Med.* 2, 1-6. 10.1186/1479-5876-2-39 15555080 PMC534783

[DEV205644C26] Newmark, P. A., Reddien, P. W., Cebria, F. and Alvarado, A. S. (2003). Ingestion of bacterially expressed double-stranded RNA inhibits gene expression in planarians. *Proc. Natl Acad. Sci. USA* 100, 11861-11865. 10.1073/pnas.1834205100 12917490 PMC304099

[DEV205644C28] Rao, X., Huang, X., Zhou, Z. and Lin, X. (2013). An improvement of the 2^∧^ (–delta delta CT) method for quantitative real-time polymerase chain reaction data analysis. *Biostat. Bioinform. Biomath.* 3, 71-85. PMC4280562 25558171

[DEV205644C29] Ravikumar, S., Devanapally, S. and Jose, A. M. (2019). Gene silencing by double-stranded RNA from C. elegans neurons reveals functional mosaicism of RNA interference. *Nucleic Acids Res.* 47, 10059-10071. 10.1093/nar/gkz748 31501873 PMC6821342

[DEV205644C30] Rivera, A. S., Hammel, J. U., Haen, K. M., Danka, E. S., Cieniewicz, B., Winters, I. P., Posfai, D., Wörheide, G., Lavrov, D. V., Knight, S. W. et al. (2011). RNA interference in marine and freshwater sponges: actin knockdown in *Tethya wilhelma* and *Ephydatia muelleri* by ingested dsRNA expressing bacteria. *BMC Biotechnol.* 11, 1-15. 10.1186/1472-6750-11-67 21679422 PMC3146823

[DEV205644C31] Rosa, C., Kamita, S. G. and Falk, B. W. (2012). RNA interference is induced in the glassy winged sharpshooter *Homalodisca vitripennis* by actin dsRNA. *Pest Manag. Sci.* 68, 995-1002. 10.1002/ps.3253 22345053

[DEV205644C32] Schumpert, C. A., Dudycha, J. L. and Patel, R. C. (2015). Development of an efficient RNA interference method by feeding for the microcrustacean Daphnia. *BMC Biotechnol.* 15, 1-13. 10.1186/s12896-015-0209-x 26446824 PMC4597761

[DEV205644C33] Shan, G. (2010). RNA interference as a gene knockdown technique. *Int. J. Biochem. Cell Biol.* 42, 1243-1251. 10.1016/j.biocel.2009.04.023 19442757

[DEV205644C34] Sheffield, J. B. (2007). ImageJ, a useful tool for biological image processing and analysis. *Microsc. Microanal.* 13, 200-201. 10.1017/S1431927607076611

[DEV205644C35] Timmons, L., Court, D. L. and Fire, A. (2001). Ingestion of bacterially expressed dsRNAs can produce specific and potent genetic interference in *Caenorhabditis elegans*. *Gene* 263, 103-112. 10.1016/S0378-1119(00)00579-5 11223248

[DEV205644C36] Wang, E. and Hunter, C. P. (2017). SID-1 functions in multiple roles to support parental RNAi in *Caenorhabditis elegans*. *Genetics* 207, 547-557. 10.1534/genetics.117.300067 28751423 PMC5629322

[DEV205644C37] Zakas, C. (2022). Streblospio benedicti: a genetic model for understanding the evolution of development and life-history. *Curr. Top. Dev. Biol.* 147, 497-521. 10.1016/bs.ctdb.2021.12.021 35337460

[DEV205644C38] Zakas, C., Deutscher, J. M., Kay, A. D. and Rockman, M. V. (2018). Decoupled maternal and zygotic genetic effects shape the evolution of development. *eLife* 7, e37143. 10.7554/eLife.37143 30198842 PMC6168281

[DEV205644C39] Zhang, Y., Kan, D., Zhou, Y., Lian, H., Ge, L., Shen, J., Dai, Z., Shi, Y., Han, C. and Yang, J. (2024). Efficient RNA interference method by feeding in *Brachionus plicatilis* (Rotifera). *Biotechnol. Lett.* 46, 961-971. 10.1007/s10529-024-03524-w 39235648

