# Supplementary material for: A methodology for RNAi gene knockdown in the marine annelid Streblospio benedicti by delivering dsRNA
Source: Development. 2026 Aug 6;153(15):dev205644. doi: 10.1242/dev.205644 (PMC13480956; doi:10.1242/dev.205644)
Supplement: Supplementary file 1 [file develop-153-205644-s1.pdf]

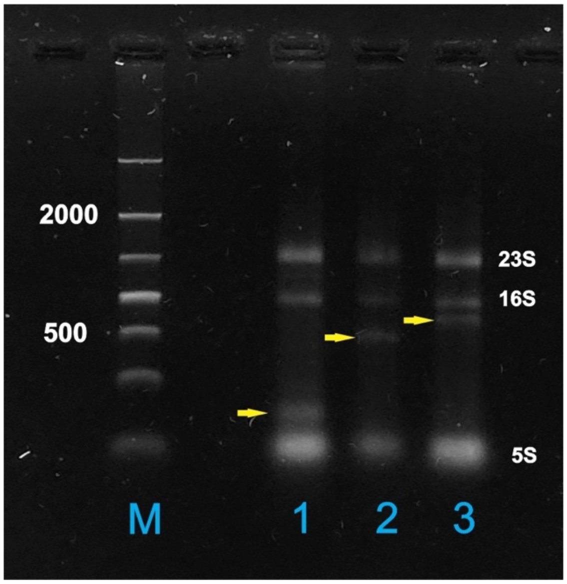

Fig. S1. dsRNA Induction. 1% agarose gel loaded with total RNA of induced bacteria. M= 3000bp DNA ladder,1= Control sample MCS (L4440 plasmid with no gene insert), 2= L4440 plasmid with a fragment of the *R-opsin* and 3 = L4440 plasmid with a fragment of the *F-actin*. The three bands in lanes 1, 2 and 3 correspond to the ribosomal RNA subunits of the bacteria (23S, 16S and 5S). Yellow arrows indicate the dsRNA expressed for each sample 4 hours after the induction with 0.4 mM IPTG.

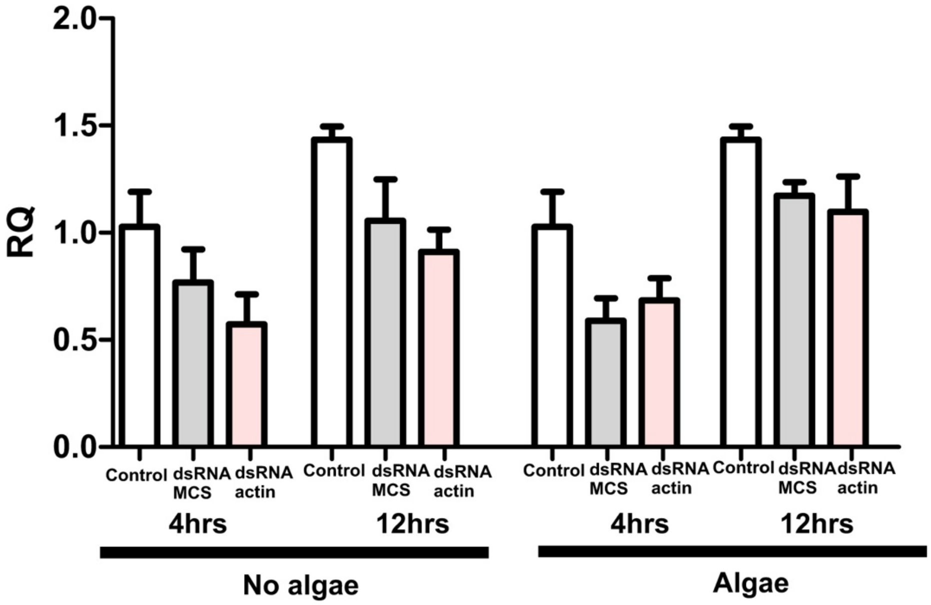

Fig. S2. Larval feeding of *F-actin* dsRNA. qPCR analysis of the *F-actin* expression levels with and without algae at two timepoints post feeding. Y-axis denotes relative levels of expression (RQ). Bars represent mean ± SE of n=3 replicates. One way analysis of variance with Tukey's multiple comparison test for each treatment were calculated and no significant statistical significance in all cases tested were determined. MCS: Multiple cloning site of the L4440 plasmid.

Development • Supplementary information

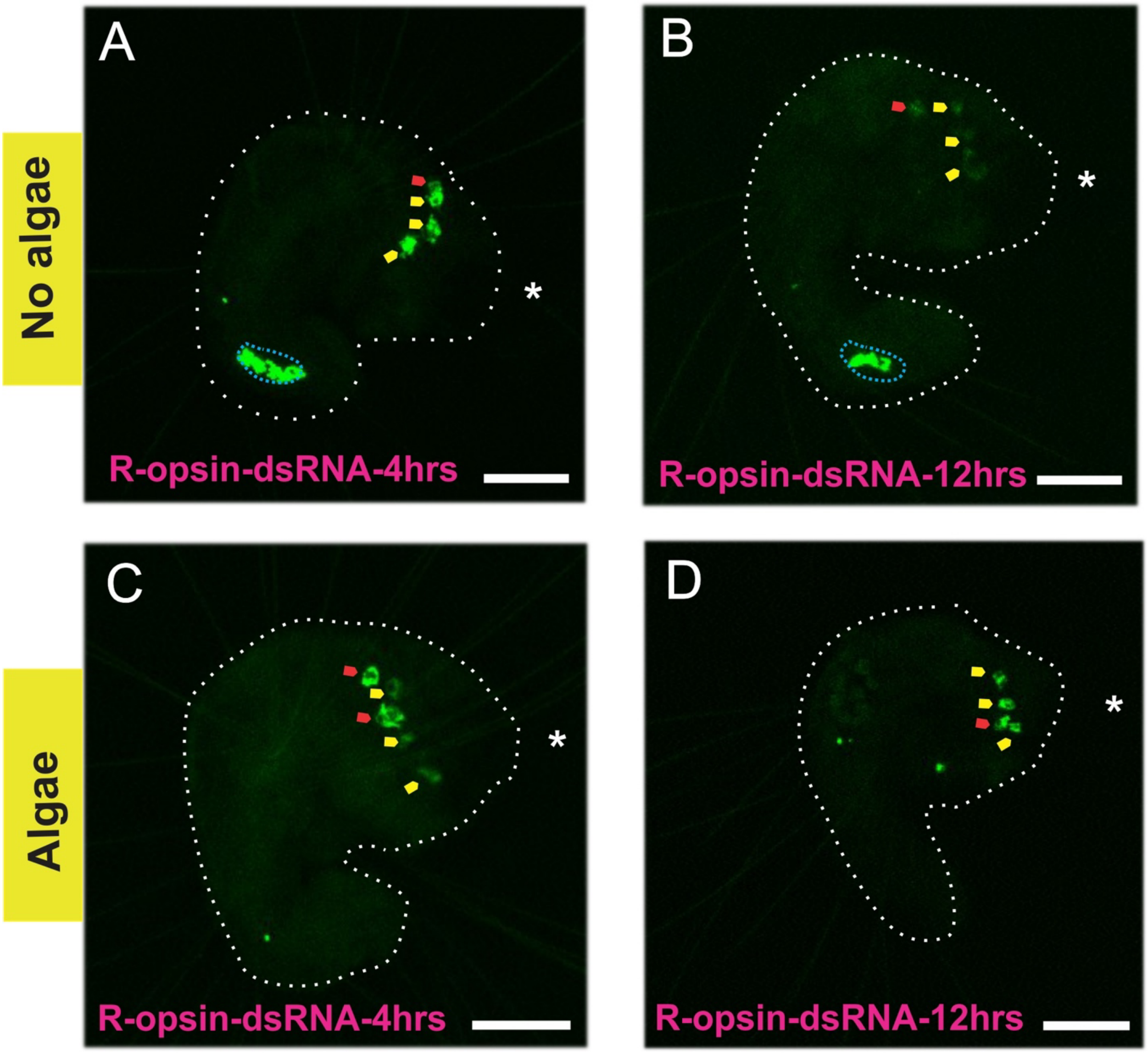

Fig. S3. HCR *in situ* hybridization of the *S. benedicti R-opsin* dsRNA larval feeding experiment. Yellow arrowheads indicate *R-opsin* domains from the right side and red arrowheads indicate *R-opsin* domains from the left side. White asterisk indicates the anterior end of the larva. White dashed lines outline the body for each individual sample. Scale bars = 45 µm. A) Early swimming larva from the *R-opsin* dsRNA /no algae/4h experiment. Lateral view shows four *R-opsin* domains— three on the right and one on the left. Blue dotted circle shows consumed bacteria expressing *R-opsin* dsRNA in the larval gut that reacted with the *R-opsin* probes. B) Early swimming larva from the *R-opsin* dsRNA /no algae/12h experiment. Lateral view shows four *Ropsin* domains— three on the right and one on the left. Blue dotted circle shows consumed bacteria expressing *R-opsin* dsRNA in the larval gut that reacted with the R-opsin probes. C) Early swimming larva from the *R-opsin* dsRNA / algae/ 4h experiment. Lateral view shows five *R-opsin* domains— three on the right and two on the left. D)Early swimming larva from the *R-opsin* d sRNA/ algae/ 12h experiment. Lateral view shows five *R-opsin* domains— three on the right and one on the left.

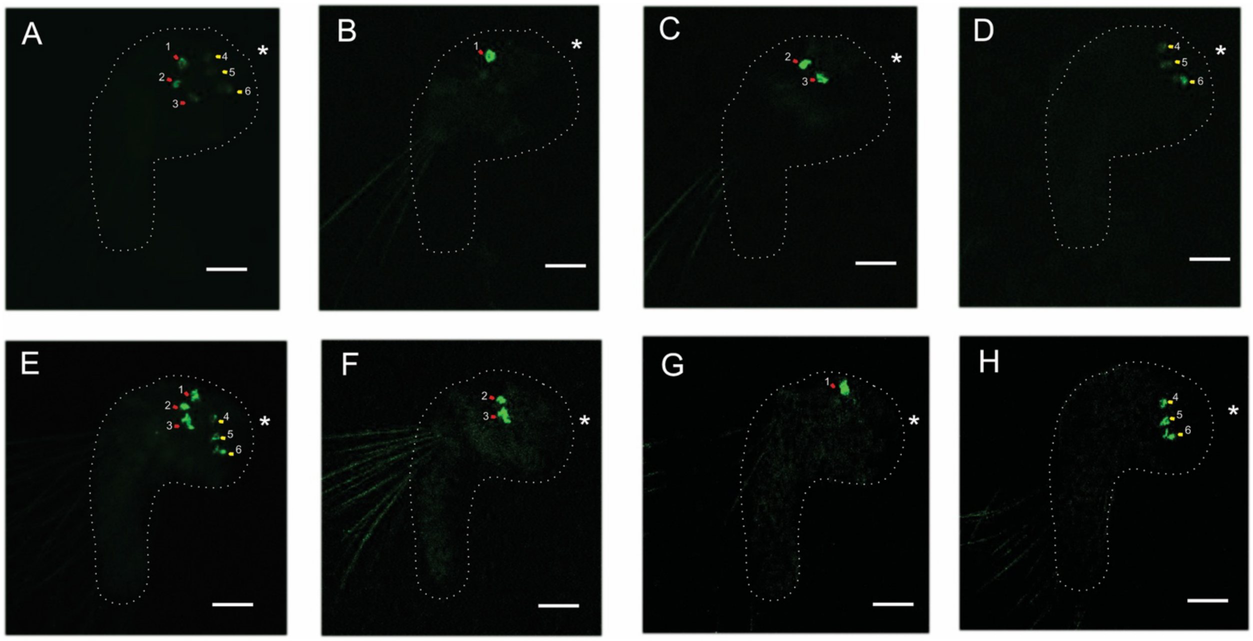

Fig. S4. HCR *in situ* hybridization of the *S. benedicti* MCS dsRNA larval feeding experiment. Yellow arrowheads indicate R-opsin domains from the right side and red arrowheads indicate R-opsin domains from the left side. White asterisk indicates the anterior end of the larva. White dashed lines outline the body for each individual sample. Scale bars = 50 µm. **Early swimming larval sample 1 (MCS dsRNA/no algae/12 hr).** A) Z-stack image (40 layers) of a maximum intensity image showing the six R-opsin domains. B) Layer number 12 of the Z-stack image showing two R-opsin domains. C)Layer number 20 of the Z-stack image showing one R-opsin domain. D) Layer number 33 showing three R-opsin domains. **Early swimming larval sample 2 (MCS dsRNA/algae/4hr).** E) Z-stack image (40 layers) of a maximum intensity image showing the six R-opsin domains. F) Layer number 9 of the Z-stack image showing two R-opsin domains. G) Layer number 28 of the Z-stack image showing one R-opsin domain. H) Layer number 36 showing three R-opsin domains.

Development • Supplementary information

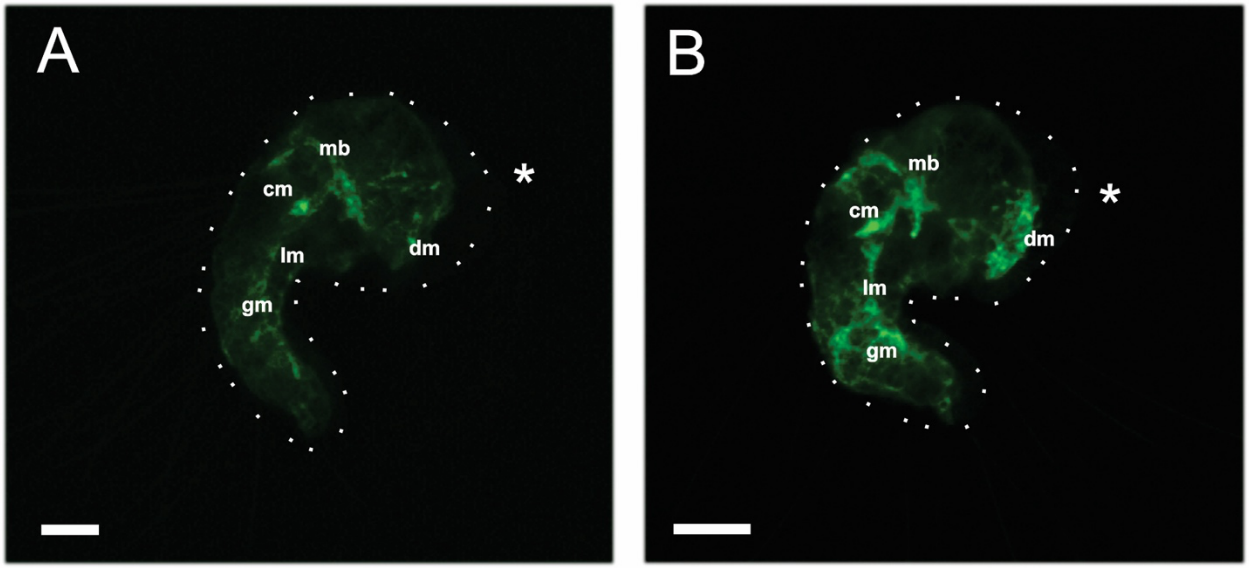

Fig. S5. HCR *in situ* hybridization of the *S. benedicti F-actin* dsRNA and MCS dsRNA larval feeding experiment. A) Lateral view of an early swimming larva from the feeding experiment showing expression of the *F-actin* gene (the sample fed control bacteria expressing dsRNA /no algae/ 4h after washing the samples). B) Lateral view of an early swimming larva from the feeding experiment showing expression of the *F-actin* gene (the sample fed bacteria expressing F-actin dsRNA /no algae/ 4h after washing the samples). *lm*=longitudinal muscle, *gm*=gastric muscle, *mb*= muscle band of the head, *cm*=chaetal muscle and *dm*=dense muscle mass of the ventral head. White asterisk indicates the anterior end of the individuals imaged. White dashed lines showed the whole body for each individual sample. Scale bars = 45 µm.

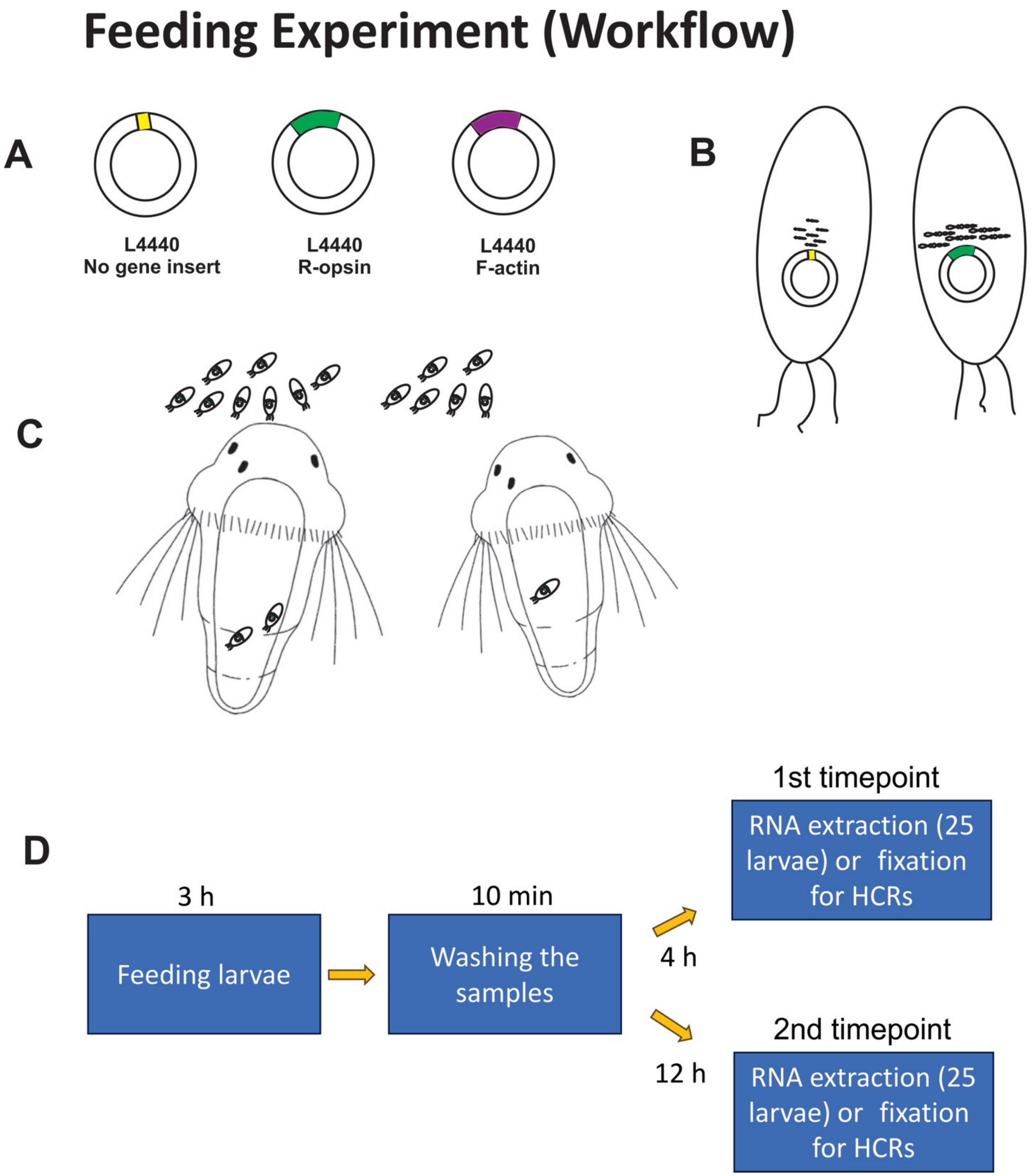

Fig. S6. dsRNA Feeding Experiment workflow and drawing. A) Drawings of the three different L4440 plasmids that were induced for the feeding experiment (L4440 no gene insert, MCS; L440 with a fragment of the *R-opsin* and L4440 with a fragment of the *F-actin)*. B) Drawings of induced bacteria expressing dsRNA in their cytoplasm. C) Drawings of early swimming larvae feeding bacteria expressing dsRNA. D) Workflow of the feeding experiment.

Development • Supplementary information

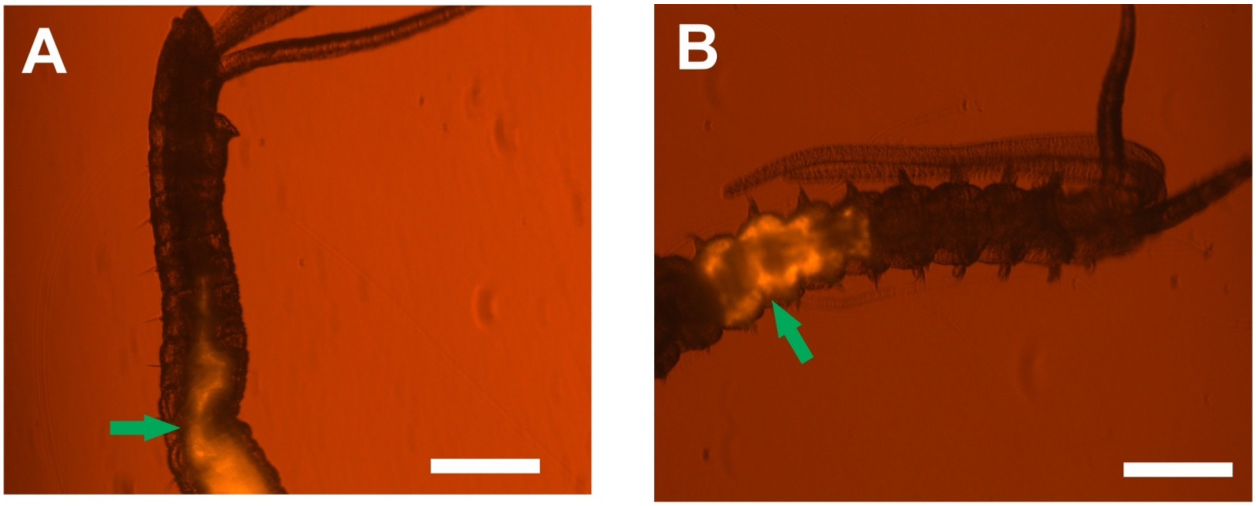

Fig. S7. Microinjection of dsRNA and dextran into 3-week-old juveniles of *S. benedicti*. A) Lateral view of an individual injected in the lumen of the gut. B) Dorsal view of an individual injected in the body cavity of two segments located five segments below the prostomium. Green arrows indicate the solution injected that contains MCS dsRNA + dextran. Scale bars= 100 µm.

Table S1. Ct values of the feeding experiment (*R-opsin*) and the ANOVA test for each treatment.

Available for download at
https://journals.biologists.com/dev/article-lookup/doi/10.1242/dev.205644#supplementary-data

Table S2. Ct values of the feeding experiment (*F-actin*) and the ANOVA test for each treatment.

Available for download at
https://journals.biologists.com/dev/article-lookup/doi/10.1242/dev.205644#supplementary-data

Table S3. Number of larvae from the feeding experiment showing four, five or six *R-opsin* domains from the HCR imaging.

	**4 R-opsin domains**	**5 R-opsin domains**	**6 R-opsin domains**	**Total**
**control 4hrs MCS**	0	0	20	20
**bacteria 4hrs R-opsin**	12	8	0	20
**control 12 hrs MCS**	0	0	20	20
**bacteria 12 hrs R-opsin**	14	6	0	20
**control 4hrs MCS algae**	0	0	20	20
**bacteria 4hrs R-opsin algae**	13	6	1	20
**control 12hrs MCS algae**	0	0	20	20
**bacteria 12hrs R-opsin algae**	11	9	0	20

Development • Supplementary information

Table S4. Ct values of the microinjection experiment (*R-opsin*) and the ANOVA test for each treatment.

Available for download at
https://journals.biologists.com/dev/article-lookup/doi/10.1242/dev.205644#supplementary-data

Table S5. Ct values of the microinjection experiment (*F-actin*) and the ANOVA test for each treatment.

Available for download at
https://journals.biologists.com/dev/article-lookup/doi/10.1242/dev.205644#supplementary-data

Table S6. DNA sequences of the *R-opsin* and *F-actin* (from the transcriptome and the gene inserted into the plasmid).

Available for download at
https://journals.biologists.com/dev/article-lookup/doi/10.1242/dev.205644#supplementary-data

Table S7. RNA concentration of the larvae from the larvae from the feeding experiment and injected juveniles from the dsRNA microinjections.

Available for download at
https://journals.biologists.com/dev/article-lookup/doi/10.1242/dev.205644#supplementary-data

Table S8. Oligonucleotides used in this study.

Name	Sequence	Comment
R-opsin FWD	TAATACGACTCACTATAGGGCCAGTACCAAGCACCTTCGGAC	Amplifying a DNA fragment from cDNA
R-opsin RS	TAATACGACTCACTATAGGGGAAGAACATAGGCACTCCAAAC	Amplifying a DNA fragment from cDNA
F-actin FWD	TAATACGACTCACTATAGGGGACGATGATGTTGCTGCTCTCG	Amplifying a DNA fragment from cDNA
F-actin RS	TAATACGACTCACTATAGGGCGCTCGGTAAGGATCTTCATCA	Amplifying a DNA fragment from cDNA
Backbone Forward	TTGGGTAACGCCAGGGTTTT	Amplifying a DNA fragment from a plasmid
Backbone Reverse	TACCGCCTTTGAGTGAGCTG	Amplifying a DNA fragment from a plasmid
*R-opsin*FW qPCR	CCTCACGGTCAACAGCATCT	Primer for qPCR
*R-opsin*RS qPCR	CTGCCTTCATGGGGTTGGAT	Primer for qPCR<br>Primer Efficiency: 92.784
*F-actin*FW qPCR	TGGTGGGTATGGGCCAGAAG	Primer for qPCR
*F-actin*RS qPCR	CCAACTGGGACGACATGGAGA	Primer for qPCR<br>Primer Efficiency: 94.682
*EF-1*FW qPCR	TGTGGAGACTTTCAGCGAGT	Primer for qPCR
*EF-1*RS qPCR	CGAGGCTCACTTCTTCTTGC	Primer for qPCR<br>Primer Efficiency: 94.238

Table S9. HCR hairpin probes of the *F-actin* gene.

Available for download at
https://journals.biologists.com/dev/article-lookup/doi/10.1242/dev.205644#supplementary-data

Table S10. HCR hairpin probes of the *R-opsin* gene.

Available for download at
https://journals.biologists.com/dev/article-lookup/doi/10.1242/dev.205644#supplementary-data
