# Supplementary material for: A methodology for RNAi gene knockdown in the marine annelid Streblospio benedicti by delivering dsRNA
Source: Development. 2026 Aug 6;153(15):dev205644. doi: 10.1242/dev.205644 (PMC13480956; doi:10.1242/dev.205644)
Supplement: Supplementary file 2 [file develop-153-205644_review_history.pdf]

A methodology for RNAi gene knockdown in the marine annelid *Streblospio benedicti* by delivering dsRNA

Jose Maria Aguilar-Camacho and Christina Zakas
DOI: 10.1242/dev.205644

Editor: Cassandra Extavour

Review timeline

Original submission:	9 March 2026
Editorial decision:	9 April 2026
First revision received:	17 June 2026
Accepted:	6 July 2026

Original submission

First decision letter

MS ID#: dev.205644

MS TITLE: A methodology for RNAi gene knockdown in the marine annelid Streblospio benedicti by delivering dsRNA

AUTHORS: Jose Maria Aguilar-Camacho and Christina Zakas

Dear Dr Zakas,

I have now received all the referees' reports on the above manuscript, and have reached a decision. The referees' comments are appended below.

As you will see, the referees express considerable interest in your work, but have some significant criticisms and recommend a substantial revision of your manuscript before we can consider publication. If you are able to revise the manuscript along the lines suggested, which may involve further experiments, I will be happy receive a revised version of the manuscript. Your revised paper will be re-reviewed by one or more of the original referees, and acceptance of your manuscript will depend on your addressing satisfactorily the reviewers' major concerns. Please also note that Development will normally permit only one round of major revision. If it would be helpful, you are welcome to contact us to discuss your revision in greater detail. Please send us a point-by-point response indicating your plans for addressing the referees' comments, and we will look over this and provide further guidance.

Please attend to all of the reviewers' comments and ensure that you upload both a 'clean' version of your Word file, along with a highlighted version clearly showing where you have made changes in the revised manuscript. Please avoid using 'Tracked changes' in Word files as these are lost in PDF conversion. I should be grateful if you would also provide a point-by-point response detailing how you have dealt with the points raised by the reviewers in the 'Response to Reviewers' box. If you do not agree with any of their criticisms or suggestions please explain clearly why this is so.

Reviewer 1

SUMMARY OF THE ADVANCE MADE IN THIS PAPER AND ITS POTENTIAL SIGNIFICANCE TO THE FIELD

© 2026. Published by The Company of Biologists under the terms of the Creative Commons Attribution License (https://creativecommons.org/licenses/by/4.0/).

This manuscript develops RNAi for mRNA knockdown in the marine annelid Streblospio benedicti, which has two developmental morphs --- a planktotrophic morph and a lecithotrophic morph. They used two separate methods for delivering double-stranded RNA; they fed bacteria expressing the dsRNA to planktotrophic larvae and injected dsRNA into juveniles. The authors attempted knockdown of two genes, R-opsin and F-actin, followed by quantification of mRNA levels by qPCR (larvae and juveniles) and HCR ISH (larvae). Studying development in this annelid has the potential to answer many interesting questions concerning life history evolution and the genetic control of larval versus adult features. The lab has already generated multiple resources for this animal including a culturing system and a high-quality, chromosome-level assembly. I think this manuscript is an important step forward in being able to test gene function in S. benedicti, and publication would benefit researchers who want to study this annelid as well as researchers who want to manipulate gene function in other understudied marine larvae. Currently, this manuscript fits as a Techniques and Resources paper (not a Research Article or Report) since it describes approaches to use RNAi in for planktotrophic larval and juvenile stages of an annelid and does not focus on understanding developmental mechanisms.

SUGGESTIONS TO AUTHORS

Main questions/comments
Overall, the manuscript needs editing for clarity and to fix grammatical mistakes. Adding more reasoning or details for why certain approaches were used would help other researchers adapt these methods for their animal.

I also would like to see more assessments of the larval phenotypes after introduction of the dsRNA (outlined below). This would help with interpretation of the success of mRNA knockdown. The approaches used resulted in knockdown of R-opsin transcript levels (qPCR), but R-opsin expression (HCR ISH) was still detected in most of the eye spots (four to five instead of six) in the head. Why is R-opsin expression only absent in a few of the eye spots on the left side of the head? Wouldn't you expect to see a loss or reduction of R-opsin in all the eye spots? This doesn't seem like a penetrant phenotype and given that the method did not have a measurable effect on F-actin in larvae, this technique may not be very effective at reducing mRNA levels for developmental genes that are expressed at higher levels or in more cells.

The approaches used in the study don't allow mRNA knockdown at larval stages in the lecithotrophic morphs since they don't feed. Did you try methods for introducing the dsRNA into the lecithotrophic larvae? Did you try introducing dsRNA into juveniles that developed from lecithotrophic larvae, or did you only inject juveniles that developed from planktotrophic larvae? This information needs to be added to section 4.3 in the methods.

Did you try feeding the juveniles bacteria expressing the dsRNA?

Additional comments
Fix verb tense throughout. When describing experiments that have already been carried out, use past tense. There are places where you switch back and forth between past and present tense.

Gene and mRNA names are generally italicized. Please fix this throughout the manuscript.

Abstract
"…..which is a model system for studying evolutionary and developmental biology that currently has no"

"We fed bacteria expressing dsRNA to early planktotrophic larvae and microinjected in vitro transcribed dsRNA into juveniles"

"The efficiency varied depending on the gene and timing, but this is typical for RNAi based approaches."

Introduction

© 2026. Published by The Company of Biologists under the terms of the Creative Commons Attribution License (https://creativecommons.org/licenses/by/4.0/).

"Hydroides elegans (Darin et al. 2025); and specifically filter-feeding invertebrates such as the oyster Crassostrea gigas (Feng et al. 2019), the marine sponge Tethya wilhema, and the freshwater sponge Ephydatia fluviatilis (Rivera et al. 2011)." Need a verb after the semi-colon.

Results and Discussion
Gene expression
The description of R-opsin expression at the beginning of section 2.1 was confusing to me. Is there one R-opsin homolog in S. benedicti? If so, please add that information and reference the gene orthology analysis. Saying that "Six R-opsin domains are expressed" makes it sound like different splice variants of R-opsin are expressed in different regions during development. I might instead say that 'R-opsin is expressed in six different spots/domains in the larval head throughout development'. The same goes for the following sentences. I would say that 'R-opsin is expressed…'.

First paragraph, section 2.1. It's not clear to me why Aquilar-Camacho et al. 2024 is being referenced here since this is paper characterizes Hox gene expression in S. benedicti. Is this a reference for the HCR technique? If so, I might move the reference to the methods.

Second paragraph, section 2.1. Is F-actin expression only detected in the muscle fibers? You describe the arrangements of the muscle fibers throughout this paragraph, but it's not clear to me if you are describing the F-actin expression. If so, I might clarify and say that "F-actin was detected in muscle fibers throughout development". Also, if you have a reference for phalloidin labeling of muscle fibers in S. benedicti, I would add that to this section.

Did you examine expression of R-opsin or F-actin in juveniles that developed from lecithotrophic larvae or only from planktotrophic larvae?

Larval feeding results
In Fig. 2, you see a reduction in R-opsin transcript levels at both 4 and 12 hours after feeding larvae the R-opsin dsRNA. Did you try any later timepoints to see how long this reduction lasts? A time course showing levels of transcript reduction including recovery of the mRNA could be helpful in interpreting the effectiveness of the knockdown. Related to this, why do you think the RNAi caused loss of R-opsin in only a subset of eyespots? Do you think feeding the larvae more bacteria or bacteria expressing higher amounts of the dsRNA would cause a more penetrant phenotype? Do you think it relates to propagation of the dsRNA between cells? Have you checked the genome of S. benedicti to look for the proteins that might be involved in this (e.g., dsRNA transporter proteins like SID-1)?

For Fig. 3, adding some labels to the figure panels would be helpful. For example, you can labels showing which panels have control animals versus which panels have animals that were fed the R-opsin dsRNA. You could also add labels to show time after feeding.

Fig. 3, Why are some larvae shown from a lateral view and some larvae shown from a dorsal view? It would be easier to interpret the results if all larvae were shown from a dorsal view. In panels B and C, which are lateral, I can't tell which spots of R-opsin expression are left versus right based on the image itself and instead must rely on the red and yellow arrowheads. I would also add the DIC images of the same animals, same view to show the pigmentation of the eyespots in addition to the R-opsin expression. This would help with interpretation of the results.

"Although no significant differences in the reduction of the expression levels for the F-actin feeding experiment to generate an actin knockdown were detected in this experiment" Awkwardly-worded sentence

Did you examine phalloidin labeling or F-actin expression by HCR in larvae that were treated with the F-actin dsRNA? I think it's important to examine the phenotypes in addition to the qPCR results. I would add data for this.

Juvenile injection results
The dsRNA was injected into both the gut lumen and into the body cavity in two different segments of juveniles. Did you try injecting into either the gut lumen or the body cavity alone? What was the

© 2026. Published by The Company of Biologists under the terms of the Creative Commons Attribution License (https://creativecommons.org/licenses/by/4.0/).

reasoning behind doing three different injection sites per animal? It would be helpful to include some discussion of this to help others trying to test RNAi methods in their animal.

Related to the point above, do you think the dsRNA is being propagated between cells after being ingested or injected into the gut (as in C. elegans), and this is how it becomes targeted to the head? You were able to see expression of R-opsin dsRNA in the gut of fed larvae (Fig. 3B), but the dsRNA didn't appear to be very wide-spread. Can you talk more about this?

For the juveniles, why didn't you do HCR after dsRNA injection? Does HCR not work in juveniles?

Methods
Section 4.3 Please add the details for the injection needles. What type of quartz glass was used (inner and outer diameter), and what parameters were used to pull the needles? I'm assuming a laser puller was used since this is quartz glass, so you should be able to include the heat, velocity, delay, etc. Alternatively, if you purchased the needles, please include the catalog number.

"The solution was injected (pi 150, ti 1.20, pc 60) in..." It's not clear to me what "pi", "ti" and "pc" refer to.

Reviewer 2

SUMMARY OF THE ADVANCE MADE IN THIS PAPER AND ITS POTENTIAL SIGNIFICANCE TO THE FIELD

Thank you for the opportunity to review this manuscript. This manuscript presents an attempt to establish RNAi-mediated gene knockdown in the marine annelid Streblospio benedicti using two post-embryonic delivery strategies: feeding dsRNA-expressing bacteria to early planktotrophic larvae and microinjecting in vitro-transcribed dsRNA into 3-week-old juveniles. The authors test two targets, R-opsin and F-actin, assess transcript reduction by qPCR in both developmental contexts, and use HCR in situ hybridization to visualize reduced larval R-opsin signal. The study is potentially important because S. benedicti is a useful evo-devo system that currently lacks functional gene manipulation tools. If the method is validated more rigorously and reported with greater procedural transparency, this work could become a valuable technical entry point for functional studies in this species. Overall, the manuscript addresses an important technical gap, but it would be substantially stronger with more rigorous validation, clearer limits on the claim, and greater methodological transparency.

SUGGESTIONS TO AUTHORS

Major comments:
Please narrow the central claim and avoid overgeneralization. At present, the data most clearly support the successful knockdown of R-opsin. In contrast, F-actin shows no significant reduction in the larval feeding experiment and only a partial effect in juveniles by microinjection at one time point, 12hrs. For this reason, the manuscript should avoid implying that RNAi is broadly established in S. benedicti as a general tool. A more defensible claim would be that the authors provide a promising stage-specific delivery system that can reduce transcript levels for some genes.

Specificity controls should be strengthened. The manuscript relies on a single dsRNA fragment per target. Please provide an off-target assessment, ideally using sequence comparisons against available transcriptome/genome resources. A second, non-overlapping dsRNA fragment for the same target would greatly strengthen the case that the observed effects are sequence-specific.

The evidence for a robust knockdown toolkit is still incomplete. The current evidence relies mainly on qPCR and, for larval R-opsin, qualitative HCR signal reduction. For a Techniques and Resources paper, stronger validation is needed. Please add quantitative tissue-level and, where feasible, functional or phenotypic validation. In particular, the manuscript would be substantially strengthened by including a figure showing histological or tissue-level analyses demonstrating whether the knockdown is accompanied by detectable morphological changes. It would also be very helpful to include a construct diagram showing how dsRNA is produced in E. coli (for example, the L4440-based construct with the target fragment and its positioning T7 promoters), together with a schematic overview of the feeding RNAi workflow. These figures would make the bacterial dsRNA-

© 2026. Published by The Company of Biologists under the terms of the Creative Commons Attribution License (https://creativecommons.org/licenses/by/4.0/).

production step, the overall experimental design, and the feeding and sampling timeline much easier for readers to visualize.

Because this is a Techniques and Resources paper, the procedural details are needed. Please report, in a table, the total number of animals treated per condition, the number surviving to analysis, the success number and its rate of injection, the proportion of animals showing measurable knockdown, and the actual injected volume per animal.

Minor comments:
"synthetized" should be "synthesized."
"HCR in situs" should be "HCR in situ."
"4-5 viable domains out of six" likely should be "4-5 visible domains out of six."
"Qbit 4 Fluorometer" should be "Qubit 4 Fluorometer."
Please check "Tethya wilhema" in the Introduction against the cited literature; it likely should be "Tethya wilhelma."
In the Figure 1 legend, panels I and J appear to be mislabeled as R-opsin. These likely should refer to F-actin.
In Figure 4, the bar plot has *** annotations, but the legend is missing. Please make the legend consistent with the plotted symbols in all figures.

<u>Reviewer 3</u>

SUMMARY OF THE ADVANCE MADE IN THIS PAPER AND ITS POTENTIAL SIGNIFICANCE TO THE FIELD

In this manuscript, authors present an RNAi approach for the marine polychaete Streblospio benedicti, a species used for developmental evolution and evolutionary genetics studies as it has two different larval forms in a single species. The authors report two different methods for dsRNA delivery, one by feed bacteria expressing dsRNA to early larvae and another method by microinject in vitro transcribed dsRNA in juvenile worms. They report both a successful and an unsuccessful case, indicating that RNAi efficiency likely depends on the target gene, developmental timing, or dsRNA dosage. Overall, this is an informative contribution that will help people who study other similar species, or life stages where temporal control of gene knock down is needed. The methods presented are written in detail and are appropriate. Parallel assessments such as in situ hybridizations and qPCRs were used. However, the manuscript will benefit from some added details, figure and text clarifications.

SUGGESTIONS TO AUTHORS

Major points
Please insert line numbering and page numbers into your pdf to make it easier for reviewers to refer to specific lines and pages in the manuscript. It takes significantly more time to review a paper without this easy addition.

Section: Results 2.3
- The authors mention quantification in the first sentence, but I don't see that for HCRs, do they mean something else? If they mean Supplement Data 8, this tab in the spreadsheet is really hard to follow. If the authors plot these numbers and integrate them into the HCR figure as quantification, that would justify the verbiage, and will do more justice to this experiment in the main figure.
- "Although no significant differences in the reduction…" - Sentence missing verb, or it has weird structure that makes it hard to follow.

Section 2.4
- "We injected in vitro transcribed dsRNA (120 ng/ µL)"
o Can the authors comment on why these particular concentrations were chosen, and whether they tried other concentrations? This would be helpful for others who want to test similar methods, since this is a methods paper.
o Did they do any initial toxicity tests with control dsRNA for what is the max non-toxic concentration that can be used? Same for larval feeding.

© 2026. Published by The Company of Biologists under the terms of the Creative Commons Attribution License (https://creativecommons.org/licenses/by/4.0/).

Figure 3 is not doing justice to the results of this paper. There are multiple issues with it:
- Labels are missing
- Number of samples not reported
- Variation in phenotypes unclear because a single image is shown, and the variation is buried into a difficult/unclear supplementary table (suppl. 8)
- Some of the ropsin spots are hard to differentiate. At least digital zooms of these can be provided, not necessarily new photos.

Results: Sections 2.2 and 2.4
- Please clarify early in the text what constitutes the control for both feeding and injection experiments.

Page 7
- "Therefore, three hours of ingesting dsRNA-expressing bacteria in early planktotrophic larvae was sufficient for the larvae to ingest bacteria and reduce Ropsin gene expression (Figure 2; Supplementary Data 2)." --- The supplementary data only includes F-actin dsRNA, not R-opsin.

Methods
Section 4.2.
- How were larvae processed for RNA extraction? Clarify whether they were pooled and how many were pooled.
- "The feeding duration was chosen based…." This sentence seems pretty important but hard to follow and vague for it to be useful to others.
o In the same sentence "under laboratory conditions": What does this mean? Isn't everything under lab conditions???
o In the same sentence "after 3h larvae died…" Unclear, were they dying because of concentration? Why did they die after 3 hours and how did this help choosing the duration for feeding? Was being in the presence of E. coli longer than 3h the issue? Is it a hard cut off, if so that seems odd. Please be more clear and specific.
- MCS: what's the base pair length of this region?
- 4h: Shouldn't this be 6h?
- In this section, can the authors clarify if they split each batch into two or did they use different replicates for different assays (HCR vs qPCR)
- I think throughout the text and also at the end of this section, it would be good to use more clear language for controls. Here the larvae they mention in the very last sentence is better to call No Treatment Controls (since MCS is a dsRNA control)

Section 4.3
- How many worms were injected per condition? How did you process them for RNA extraction, did you pool individuals?
- "such as early juveniles and swimming lecithotrophic larvae" -- does this mean earlier than 2 weeks? Hard to know for people not familiar with this organism

Section 4.4
- It is important to report the primer efficiency for each primer set. If the authors have this, please indicate.
- Additionally, please include information about the RNA quality obtained in the experiments.
- "Three independent repeats for each replicate were examined and the samples were subjected" -- Can the authors clarify if this means they did qPCRs 3x for same samples? Or whether they had 3 replicates of the same sample per qPCR run?

Figures
Figure 1
- Please Indicate the number/ percentage of observations supporting the described phenotype.
- Please also outline the shape of the larva with a dashed line.
- Please add titles for each row group (A-E) and (F-J). It is difficult to go back and forth between the legend and images. Example: A-E rows: R-opsin F-J rows: F-actin

© 2026. Published by The Company of Biologists under the terms of the Creative Commons Attribution License (https://creativecommons.org/licenses/by/4.0/).

Figure 3
- Similarly, the figure would be clearer if row titles and the number/ percentage of observations supporting phenotypes were included (see comment on Figure 1). labels for control vs ropsin dsRNA etc. 4 vs 12 h.
- It would be really helpful to show some more examples of phenotypes in the supplementary. When the larvae were missing eyes, how were they categorized in terms of the phenotype? If one eye was missing vs 2 vs 3? supple 8 data is confusing, as there is not much explanation for it. It will be better to integrate those quantifications here, and also show some different phenotype examples in a supplementary figure

Figures 2 and 4:
- Please specify the number of samples analyzed. Did you have three replicates per sample, or three biological replicates in total? If only three biological replicates were used, it would be advisable to increase the number of experiments if possible. The limited sample size may explain why no differences were observed for F-actin.

Figure Supplement 3
- Please, mention the number or percentage of observations supporting phenotype.

Minor points
Section: Results 2.1
It would be clearer to organize the text following the developmental sequence (as shown in the figure): from the trochophore to the juvenile stage.

Figure 2: Consider spelling out what MCS is in figure legend
Figure 4: Figure itself is missing A and B labels
Figure legends of Suppl Data text: "five o six R-opsin" typo

First revision

<u>Author response to reviewers' comments</u>

A methodology for RNAi gene knockdown in the marine annelid *Streblospio benedicti* by delivering dsRNA
We thank the reviewers for their thoughtful comments which have greatly improved the manuscript. We made many clarifications throughout the paper to better illustrate the RNAi knockdown. In particular, we added more information about our phenotyping and HCR *in situ* visualization, including drawings for larval orientation. We also added additional figures in the supplement to illustrate the location of eyespots. We have included more information about possible off-target effects (we do not see evidence of this) and efficiency (we make recommendations about optimization). Our clarifications should address the potential confusion about phenotypic results: we do see a phenotypic reduction of r-opsin using HCR *in situs* and qPCR, and while we do not see a phenotype for F-actin, we do see a significant reduction in expression with qPCR. These methods are consistent with other marine invertebrate RNAi methodologies in the literature.

We also emphasized throughout the manuscript that we are presenting a technique that leads to RNA knockdown in this emerging model. The reviewers suggested many ways that the efficiency of the knockdown could be changed, such as trying additional time points, RNA quantities, sequences, etc. While we agree that this is worthwhile when trying to optimize efficiency, the results will vary across targets; meaning that these factors will have to be optimized by researchers for every gene target used. We felt that while we could try and report more combinations of variables, we have definitively shown that the RNAi knockdown methods presented here work, and this establishes the utility of RNAi in this species.

© 2026. Published by The Company of Biologists under the terms of the Creative Commons Attribution License (https://creativecommons.org/licenses/by/4.0/).

Reviewer 1: SUMMARY OF THE ADVANCE MADE IN THIS PAPER AND ITS POTENTIAL SIGNIFICANCE TO THE FIELD

This manuscript develops RNAi for mRNA knockdown in the marine annelid Streblospio benedicti, which has two developmental morphs --- a planktotrophic morph and a lecithotrophic morph. They used two separate methods for delivering double-stranded RNA; they fed bacteria expressing the dsRNA to planktotrophic larvae and injected dsRNA into juveniles. The authors attempted knockdown of two genes, R-opsin and F-actin, followed by quantification of mRNA levels by qPCR (larvae and juveniles) and HCR ISH (larvae). Studying development in this annelid has the potential to answer many interesting questions concerning life history evolution and the genetic control of larval versus adult features. The lab has already generated multiple resources for this animal including a culturing system and a high-quality, chromosome-level assembly. I think this manuscript is an important step forward in being able to test gene function in S. benedicti, and publication would benefit researchers who want to study this annelid as well as researchers who want to manipulate gene function in other understudied marine larvae. Currently, this manuscript fits as a Techniques and Resources paper (not a Research Article or Report) since it describes approaches to use RNAi in for planktotrophic larval and juvenile stages of an annelid and does not focus on understanding developmental mechanisms.

SUGGESTIONS TO AUTHORS

Main questions/comments
Overall, the manuscript needs editing for clarity and to fix grammatical mistakes. Adding more reasoning or details for why certain approaches were used would help other researchers adapt these methods for their animal.

I also would like to see more assessments of the larval phenotypes after introduction of the dsRNA (outlined below). This would help with interpretation of the success of mRNA knockdown. The approaches used resulted in knockdown of R-opsin transcript levels (qPCR), but R-opsin expression (HCR ISH) was still detected in most of the eye spots (four to five instead of six) in the head. Why is R-opsin expression only absent in a few of the eye spots on the left side of the head? Wouldn't you expect to see a loss or reduction of R-opsin in all the eye spots? This doesn't seem like a penetrant phenotype and given that the method did not have a measurable effect on F-actin in larvae, this technique may not be very effective at reducing mRNA levels for developmental genes that are expressed at higher levels or in more cells.

As the reviewer indicated we did not see a phenotype for F-actin, however we did see a phenotype for R-opsin. We agree that this may mean RNAi may have variable effects depending on the target gene used. We added three things to the manuscript: 1) We clarified that the HCRs show a reduction in R-opsin domains, which is a phenotype change (Lines 131-154) and 2) we indicated that the knockdown efficiency from our RNAi methods may be variable based on the target. (Lines 162-173). 3) We discuss why we may see a reduction in just the newly forming R-opsin domains (Lines 148-154).

The approaches used in the study don't allow mRNA knockdown at larval stages in the lecithotrophic morphs since they don't feed. Did you try methods for introducing the dsRNA into the lecithotrophic larvae? Did you try introducing dsRNA into juveniles that developed from lecithotrophic larvae, or did you only inject juveniles that developed from planktotrophic larvae? This information needs to be added to section 4.3 in the methods.

For the feeding experiment, we only use the planktotrophic larval morph which is an obligately feeding and swimming larva that develops over weeks in the plankton, while the lecithotrophic larval morph is facultatively (non-obligately) feeding with an abbreviated larval phase. This information is in the introduction. Lines 58-64. We tried to inject dsRNA into the gut of the swimming lecithotrophic larvae, but after the injection the solution was leaking. For the microinjection experiment, we only use lecithotrophic juveniles but have no reason to expect it would be different for planktotrophic juveniles. This information is indicated in the methods section. Lines 260-267.

Did you try feeding the juveniles bacteria expressing the dsRNA?

© 2026. Published by The Company of Biologists under the terms of the Creative Commons Attribution License (https://creativecommons.org/licenses/by/4.0/).

We did not try to do that because larvae are non-discriminate feeders, but juveniles eat particulate mud and have stronger preferences. That was the reason for us to inject the dsRNA in juveniles as an alternative method to deliver dsRNA.

Additional comments
Fix verb tense throughout. When describing experiments that have already been carried out, use past tense. There are places where you switch back and forth between past and present tense.

We fixed the verbs throughout the manuscript.

Gene and mRNA names are generally italicized. Please fix this throughout the manuscript.

Changes were made in the manuscript. All the genes and mRNA names are italicized.

Abstract
"…..which is a model system for studying evolutionary and developmental biology that currently has no"

We modified this sentence in the updated version of the manuscript. Lines 56-58

"We fed bacteria expressing dsRNA to early planktotrophic larvae and microinjected in vitro transcribed dsRNA into juveniles"

We modified this sentence in the updated version of the manuscript. Lines 17-18.

"The efficiency varied depending on the gene and timing, but this is typical for RNAi based approaches."

We modified this sentence in the updated version of the manuscript. Lines 21-23.

Introduction
"Hydroides elegans (Darin et al. 2025); and specifically filter-feeding invertebrates such as the oyster Crassostrea gigas (Feng et al. 2019), the marine sponge Tethya wilhema, and the freshwater sponge Ephydatia fluviatilis (Rivera et al. 2011)." Need a verb after the semi-colon.

We modified this sentence in the updated version of the manuscript. Lines 52-55.

Results and Discussion
Gene expression
The description of R-opsin expression at the beginning of section 2.1 was confusing to me. Is there one R-opsin homolog in S. benedicti? If so, please add that information and reference the gene orthology analysis. Saying that "Six R-opsin domains are expressed" makes it sound like different splice variants of R-opsin are expressed in different regions during development. I might instead say that 'R-opsin is expressed in six different spots/domains in the larval head throughout development'. The same goes for the following sentences. I would say that 'R-opsin is expressed…'.

We modified this sentence. There is one copy of r-opsin in the genome (Lines 215-216) We added “R-opsin is expressed in six different spots/domains in the larval head” Lines 87-89.

First paragraph, section 2.1. It's not clear to me why Aquilar-Camacho et al. 2024 is being referenced here since this is paper characterizes Hox gene expression in S. benedicti. Is this a reference for the HCR technique? If so, I might move the reference to the methods.

Yes, it is referring to the HCR methods. We moved this to the methods.

Second paragraph, section 2.1. Is F-actin expression only detected in the muscle fibers? You describe the arrangements of the muscle fibers throughout this paragraph, but it's not clear to me if you are describing the F-actin expression. If so, I might clarify and say that "F-actin was detected in muscle fibers throughout development".

© 2026. Published by The Company of Biologists under the terms of the Creative Commons Attribution License (https://creativecommons.org/licenses/by/4.0/).

We modified this sentence in the updated version of the manuscript. We added "F-actin was detected in muscle fibres throughout development in the planktotrophic mode of *S. benedicti*. Lines 92-93.

Also, if you have a reference for phalloidin labeling of muscle fibers in *S. benedicti*, I would add that to this section.

We do not present it here, but we used phalloidin staining in the swimming stage and early juveniles. Phalloidin is the same as the F-actin HCR expression pattern, but we wanted to see if the RNA was reduced using HCR. Since there is no visual difference in the F-actin knockdown we did not present both sets of images as they would be redundant. For reference, there are images of phalacidin staining showing muscle bands in the swimming larvae of *Streblospio benedicti* by Pernet and McHugh, 2010.

Pernet, B., & McHugh, D. (2010). Evolutionary changes in the timing of gut morphogenesis in larvae of the marine annelid Streblospio benedicti. Evolution & development, 12(6), 618-627.

Did you examine expression of R-opsin or F-actin in juveniles that developed from lecithotrophic larvae or only from planktotrophic larvae?

We only examined the expression of R-opsin and F-actin in juveniles from the planktotrophic mode. It is indicated in the methods section Lines 260-262.

Larval feeding results
In Fig. 2, you see a reduction in R-opsin transcript levels at both 4 and 12 hours after feeding larvae the R-opsin dsRNA. Did you try any later timepoints to see how long this reduction lasts?

We decided only to test gene expression differences at two time points (4 and 12 hours after feeding). We did not test later timepoints. We did add an acknowledgement that when using these methods, additional timepoint could be used to establish maximum efficiency for the particular target gene (Lines 162-166; 202-208)

A time course showing levels of transcript reduction including recovery of the mRNA could be helpful in interpreting the effectiveness of the knockdown. Related to this, why do you think the RNAi caused loss of R-opsin in only a subset of eyespots?

We agree with this, but we chose timepoints based on those used in other model systems and they did show a reduction. We added that we hypothesize that the reduction in R-opsin domains is only happening in the newly formed eyespots due to the developmental timing. We cannot verify this, as it's only speculation, nonetheless that is the pattern. It could be that RNAi is reducing the newly made transcripts and not the older ones that were previously transcribed, making up the older eyespots. We added this to the discussion Lines 148-154.

Do you think feeding the larvae more bacteria or bacteria expressing higher amounts of the dsRNA would cause a more penetrant phenotype?

Not necessarily. Small amounts of dsRNA are effective in *C. elegans* to produce a gene knockdown (Kamath et al. 2000). We are also not sure how we could control the amount of dsRNA the bacteria are producing. It is possible that higher doses of dsRNA could potentially increase the reduction of the expression levels of a particular gene in *S. benedicti*. We added a sentence about this in the document: Lines 166-173.

Kamath, R. S., Martinez-Campos, M., Zipperlen, P., Fraser, A. G., & Ahringer, J. (2000). Effectiveness of specific RNA-mediated interference through ingested double-stranded RNA in Caenorhabditis elegans. Genome biology, 2(1), research0002-1.

© 2026. Published by The Company of Biologists under the terms of the Creative Commons Attribution License (https://creativecommons.org/licenses/by/4.0/).

Do you think it relates to propagation of the dsRNA between cells? Have you checked the genome of S. benedicti to look for the proteins that might be involved in this (e.g., dsRNA transporter proteins like SID-1)?

Yes! *S. benedicti* has one gene copy for DICER and one gene copy for SID. We can only speculate about the role of Sid-1 in dsRNA uptake. In other models such as C. elegans, Sid-1 is necessary for cellularization of the dsRNA, but in insects like *Tribolobium* Sid-1 genes are present but not involved in dsRNA uptake. However, *Tribolium* maintains efficient RNAi knockdowns while other insects like *Drosophila* have neither Sid-1 genes nor efficient RNAi. Therefore, it seems the role of Sid-1 in cellular propagation is variable across invertebrates and we have not verified its role in *S. benedicti*. We now mention the presence of Sid genes in the manuscript. Lines 170-174.

For Fig. 3, adding some labels to the figure panels would be helpful. For example, you can labels showing which panels have control animals versus which panels have animals that were fed the R-opsin dsRNA. You could also add labels to show time after feeding.

We modified the figure and added labels showing which panels were fed with MCS dsRNA and R-opsin dsRNA as well as the two timepoints. We also added a drawing to show larval orientation and the R-opsin domains and eyespot orientation in both dorsal and lateral views.

Fig. 3, Why are some larvae shown from a lateral view and some larvae shown from a dorsal view? It would be easier to interpret the results if all larvae were shown from a dorsal view. In panels B and C, which are lateral, I can't tell which spots of R-opsin expression are left versus right based on the image itself and instead must rely on the red and yellow arrowheads. I would also add the DIC images of the same animals, same view to show the pigmentation of the eyespots in addition to the R-opsin expression. This would help with interpretation of the results.

Some of the larvae are shown in ventral view to show the reduction of the R-opsin domains in samples that were fed R-opsin dsRNA. Larvae that were fed MCS dsRNA are shown in either dorsal or lateral view. Red and Yellow Arrowheads indicate the right and left R-opsin domains respectively. We now include in the supplement material a Z-stack image (maximum projection image) of two wild type larvae showing the six R-opsin domains at different focal planes. We also included images of selected layers of the Z-stack showing individuals or groups of R-opsin domains corresponding to the left or right side.

We added a drawing to Fig 3 to illustrate where the eyespots and eyes occur in both the lateral and dorsal view for clarity.

We did not include the DIC images because the pigmented eye spots are not visible after the PFA fixation.

"Although no significant differences in the reduction of the expression levels for the F-actin feeding experiments to generate an actin knockdown were detected in this experiment" Awkwardly-worded sentence.

We changed that sentence for the following one which is clearer. “No significant differences were detected in the expression levels for the F-actin feeding experiment (Figure S2)”, Lines 152-155.

Did you examine phalloidin labeling or F-actin expression by HCR in larvae that were treated with the F-actin dsRNA? I think it's important to examine the phenotypes in addition to the qPCR results. I would add data for this.

We did HCRs of F-actin of larvae that fed bacteria expressing F-actin dsRNA, but no visible differences were detected. We included these figures in the supplement material.

Juvenile injection results

The dsRNA was injected into both the gut lumen and into the body cavity in two different segments of juveniles. Did you try injecting into either the gut lumen or the body cavity alone? What was the reasoning behind doing three different injection sites per animal? It would be helpful to include some discussion of this to help others trying to test RNAi methods in their animal.

© 2026. Published by The Company of Biologists under the terms of the Creative Commons Attribution License (https://creativecommons.org/licenses/by/4.0/).

We wanted to target the most tissues possible for maximal results, that was the reason that each animal was injected in both the gut lumen and the body cavity alone. Lines 282-283.

Related to the point above, do you think the dsRNA is being propagated between cells after being ingested or injected into the gut (as in C. elegans), and this is how it becomes targeted to the head? You were able to see expression of R-opsin dsRNA in the gut of fed larvae (Fig. 3B), but the dsRNA didn't appear to be very wide-spread. Can you talk more about this?

Injected dsRNA enters surrounding somatic and germline cells from the extracellular fluid via the transmembrane channel protein Sid-1. Sid-1 acts as a passive importer, allowing dsRNA to move between cells in *C. elegans* (Ravikumar et al. 2019). We do not know how dsRNA is transported to the cells expressing R-opsin in *S. benedicti* but perhaps it is the same propagation system such as: *C elegans*. Lines 169-173.

Ravikumar, S., Devanapally, S., & Jose, A. M. (2019). Gene silencing by double-stranded RNA from *C. elegans* neurons reveals functional mosaicism of RNA interference. *Nucleic Acids Research*, *47*(19), 10059-10071.

For the juveniles, why didn't you do HCR after dsRNA injection? Does HCR not work in juveniles?

HCRs do work in early developmental stages as they are whole mounts, but we are still optimizing this protocol in late developmental stages. Juveniles likely need to be sectioned for microscopy.

Methods
Section 4.3 Please add the details for the injection needles. What type of quartz glass was used (inner and outer diameter), and what parameters were used to pull the needles? I'm assuming a laser puller was used since this is quartz glass, so you should be able to include the heat, velocity, delay, etc. Alternatively, if you purchased the needles, please include the catalog number.
Quartz glass needles (QF100-70-10; Sutter Instruments) that were pulled using a micropipette puller (P-2000; Sutter Instruments). The pull settings are specific to the machine as needle pullers are variable and need to be optimized individually. Most other papers using microinjections tend to omit specific pull programs due to the mechanical variability. Lines 272-276.

"The solution was injected (pi 150, ti 1.20, pc 60) in…" It's not clear to me what "pi", "ti" and "pc" refer to.

These are the settings of the femptojet microinjector that were used to inject dsRNA in juveniles. Injection pressure 150, injection time 1.20, compensation pressure 6. Lines 281-283

Reviewer 2: SUMMARY OF THE ADVANCE MADE IN THIS PAPER AND ITS POTENTIAL SIGNIFICANCE TO THE FIELD

Thank you for the opportunity to review this manuscript. This manuscript presents an attempt to establish RNAi-mediated gene knockdown in the marine annelid Streblospio benedicti using two post-embryonic delivery strategies: feeding dsRNA-expressing bacteria to early planktotrophic larvae and microinjecting in vitro-transcribed dsRNA into 3-week-old juveniles. The authors test two targets, R-opsin and F-actin, assess transcript reduction by qPCR in both developmental contexts, and use HCR in situ hybridization to visualize reduced larval R-opsin signal. The study is potentially important because S. benedicti is a useful evo-devo system that currently lacks functional gene manipulation tools. If the method is validated more rigorously and reported with greater procedural transparency, this work could become a valuable technical entry point for functional studies in this species. Overall, the manuscript addresses an important technical gap, but it would be substantially stronger with more rigorous validation, clearer limits on the claim, and greater methodological transparency.

SUGGESTIONS TO AUTHORS

Major comments:

© 2026. Published by The Company of Biologists under the terms of the Creative Commons Attribution License (https://creativecommons.org/licenses/by/4.0/).

Please narrow the central claim and avoid overgeneralization. At present, the data most clearly support the successful knockdown of R-opsin. In contrast, F-actin shows no significant reduction in the larval feeding experiment and only a partial effect in juveniles by microinjection at one time point, 12hrs. For this reason, the manuscript should avoid implying that RNAi is broadly established in S. benedicti as a general tool. A more defensible claim would be that the authors provide a promising stage-specific delivery system that can reduce transcript levels for some genes.

We changed the emphasis throughout the manuscript to show that we have established a stage-specific tool that can have variable results depending on the target gene, but that ultimately can lead to an effective gene knockdown. We changed the title as well to reflect that this is a methodology paper.

Specificity controls should be strengthened. The manuscript relies on a single dsRNA fragment per target. Please provide an off-target assessment, ideally using sequence comparisons against available transcriptome/genome resources.

While we cannot guarantee off-target effects, we detect no additional phenotypes that would suggest other genes are affected. Notably, other recent RNAi method papers (*Hydroides* PNAS 2025) do not report any validation of off-target effects.

A BLAST alignment of the two sequences of our two dsRNA target sequences (R-opsin and F-actin) were aligned to the genome and indicate possible off-targets.

For R-opsin (467bp). The top hit on CH11 is the R-opsin gene. There are no off target matches indicated.

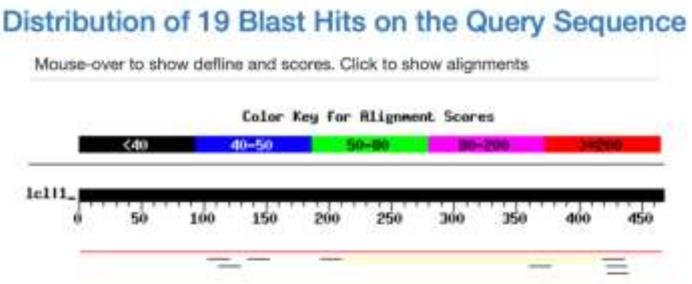

Sequences producing significant alignments:	Score (bits)	E Value
Ch_11	299	2e-79
Scaffold_371	36	4.0
Ch_8	36	4.0
Ch_7	36	4.0
Ch_6	36	4.0
Ch_3	36	4.0
Ch_2	36	4.0
Ch_10	36	4.0

Metazoan species contain different or multiple actin genes (Sebe-Pedros et al. 2013), yet a single dsRNA of the target gene can be sufficient to generate knockdown F-actin with a detectable phenotype (using Phalloidin staining or *in situ* hybridization and qPCR (Rivera et al. 2019). This gene often has many copies, but we selected it in our study because of its use in other marine invertebrate proof-of-principle papers. F-actin has been used in sponges (Rivera et al. 2019) and cnidarians (Dunn et al. 2007; Kislik et al. 2024) and assessment of off-target effects were not reported.
For *S. benedicti* F-actin (602 bp), there were six hits in different chromosomes (based on a BLAST search in the genome).

© 2026. Published by The Company of Biologists under the terms of the Creative Commons Attribution License (https://creativecommons.org/licenses/by/4.0/).

Sebé-Pedrós, A., Burkhardt, P., Sánchez-Pons, N., Fairclough, S. R., Lang, B. F., King, N., & Ruiz-Trillo, I. (2013). Insights into the origin of metazoan filopodia and microvilli. Molecular Biology and Evolution, 30(9), 2013-2023.

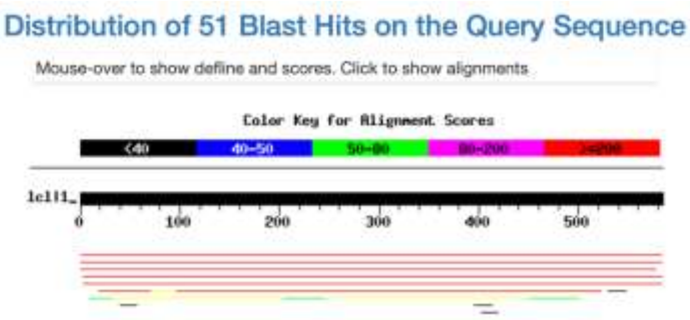

Sequences producing significant alignments:	Score (bits)	E Value
Ch_9	547	e-154
Ch_11	450	e-124
Ch_6	414	e-114
Ch_2	345	4e-93
Ch_3	329	2e-88
Ch_5	222	4e-56
Ch_10	56	5e-06
Ch_1	38	1.3
Scaffold_77	36	5.1
Ch_8	36	5.1
Ch_7	36	5.1

A second, non-overlapping dsRNA fragment for the same target would greatly strengthen the case that the observed effects are sequence-specific.

RNAi efficiency is known to be variable across many organisms depending on the specific dsRNA sequence used, and there are a wide variety of tools that have been developed to optimize sequences (for example dsRIP in insects shows the variable responses to different sequence selection). Given this issue, researchers knocking down a particular gene could try different sequences and compare efficiency to optimize. For our purposes, we are aiming for a proof-of-concept, not necessarily to show the optimal sequence for knocking down our focal genes. We clarify throughout the paper that we are presenting a technique, and we are not making any conclusions about the optimal sequence to use for our genes. (We have changed the title as well). We now include a recommendation that, given the differences we see between F-actin and R-opsin efficiency, methods should be optimized specifically for the target gene. If we were to include different dsRNA sequences, we may see a change of efficiency in our phenotypes, however, it would not eliminate the need for researchers to do this independently for their own focal genes in the future. For many of the other marine invertebrate method papers we cited (hydroides, oysters, sponges listed below) they did not show multiple fragments of the same gene to establish RNAi knockdowns (Cedden et al. 2025).

Cedden, D., Güney, G., Rostás, M., & Bucher, G. (2025). Optimizing dsRNA sequences for RNAi in pest control and research with the dsRIP web platform. BMC biology, 23(1), 114.

The evidence for a robust knockdown toolkit is still incomplete. The current evidence relies mainly on qPCR and, for larval R-opsin, qualitative HCR signal reduction. For a Techniques and Resources paper, stronger validation is needed.

© 2026. Published by The Company of Biologists under the terms of the Creative Commons Attribution License (https://creativecommons.org/licenses/by/4.0/).

In similar papers introducing RNAi to marine models the researchers used only these approaches. In oysters they validated with only qPCR and phenotypic results (Feng et al. 2019); in marine annelids (hydroides) they used qPCR, HCRs or IHC with phenotypic validation (Darin et al. 2025); and in sponges they only use qPCR, and HCRs or IHC (Rivera et al. 2019).

Some confusion may stem from phenotypic validation in our system: because r-opsin spots are not a visible phenotype, the only way to phenotypically validate a knock down is using RNA probes (HCR), which we show. We clarify this throughout the manuscript. We present the combination of 1) **phenotypic** results using HCR and 2) **qPCR** that shows the expression has been reduced. qPCR uses a housekeeping gene (in our case EF1a) to normalize, indicating the change is only in the focal gene. Our controls also demonstrate that RNA expression is reduced in the treatments only. We think this is a robust demonstration of RNA knockdown on par with other methods papers.

Please add quantitative tissue-level and, where feasible, functional or phenotypic validation. In particular, the manuscript would be substantially strengthened by including a figure showing histological or tissue-level analyses demonstrating whether the knockdown is accompanied by detectable morphological changes.

We believe that we have shown phenotypic validation of R-opsin gene knockdown using HCR. R-opsin does not affect eye development, so knocking it down would not produce a visible phenotype. It does, however, produce a phenotype in the R-opsin domains which are visible with *in situ* hybridization. F-actin knock-down does not produce a visible phenotypic difference even in the one stage where it is significantly reduced in qPCR after microinjection (12h). These are the phenotypes we reported as our results.

We can clearly see the gene expression in eyes using Whole Mount FISH, and tissue-sectioning histology is not necessary to demonstrate that the expression is only in these areas. The confocal z-stacks show whole mounts of the larvae where expression of the gene is occurring. We added a breakdown by focal plane of the confocal images to the supplement (Fig Supplement 4). Given that R-opsin is only expressed in eyespots, that is the only tissue we would query in more detail. (Our current figures show the whole body *in situ* mounts and there is no other expression of the gene in the body). However, we see no change in the phenotype of the larvae in the eyes using microscopy. It is possible that histology could show some changes in eye formation, but we do not see this in the stacks of our whole mount confocal imaging. We have not specifically quantified the reduction in expression level using fluorescence measurements because we could see whole R-opsin domains were missing, which is a clear indication of the reduction of expression. We added to the supplement additional Z-stacks to see the R-opsin domains (Figure Supplement 4).
F-actin is present throughout the body and from our fluorescent labeling we do not see any noticeable change in any body section when comparing the controls and treatment larvae. If we are understanding correctly, tissue-specific histology could show some slight reduction in F-actin formation in particular areas, but it is unlikely given that we do not see this in our confocal z-stack images. Histology of any kind has not been previously done in these larvae and would be a large undertaking for potentially minor differences in results. Unfortunately, tissue-specific RNAi is not yet possible in our system as this paper represents the first step. Similar papers introducing RNAi in marine invertebrates do not include tissue specific analyses or histology.

It would also be very helpful to include a construct diagram showing how dsRNA is produced in E. coli (for example, the L4440-based construct with the target fragment and its positioning T7 promoters), together with a schematic overview of the feeding RNAi workflow. These figures would make the bacterial dsRNA-production step, the overall experimental design, and the feeding and sampling timeline much easier for readers to visualize.
We added a figure about the feeding RNAi workflow in the supplementary material (Figure Supplement 6).

Because this is a Techniques and Resources paper, the procedural details are needed. Please report, in a table, the total number of animals treated per condition, the number surviving to analysis, the success number and its rate of injection, the proportion of animals showing measurable knockdown, and the actual injected volume per animal.

© 2026. Published by The Company of Biologists under the terms of the Creative Commons Attribution License (https://creativecommons.org/licenses/by/4.0/).

We used 25 larvae for the feeding experiment, and this information is indicated in the methods sections Lines 237-240. All the larvae survived after the feeding experiment so there was no mortality. The total number of observations supporting phenotypes for each treatment and timepoint are reported in Table S3 (based on HCR imaging). All the injected animals survived the microinjections and there was no mortality. The phenotype for the injection was indicated in the graphs for the gene knockdown (Table S4 and S5). The total amount of injected dsRNA was 120 ng/ μL dsRNA of the target gene (either R-opsin or F-actin) or MCS dsRNA and it is indicated in the methods section Lines-280-283.

Minor comments:
"synthetized" should be "synthesized."
"HCR in situs" should be "HCR in situ."
"4-5 viable domains out of six" likely should be "4-5 visible domains out of six."
"Qbit 4 Fluorometer" should be "Qubit 4 Fluorometer."
Please check "Tethya wilhema" in the Introduction against the cited literature; it likely should be "Tethya wilhelma."
In the Figure 1 legend, panels I and J appear to be mislabeled as R-opsin. These likely should refer to F-actin.
We corrected the typo mistakes in the updated version of the manuscript. P2 L40, P5 L140, P5 L143, P11 L303, P2 L52, P16 L493-494.

In Figure 4, the bar plot has *** annotations, but the legend is missing. Please make the legend consistent with the plotted symbols in all figures.

We added the P value to the figure legend of Figure 4. ***= $P < 0.0001$. P 19, L 586-587.

Reviewer 3: SUMMARY OF THE ADVANCE MADE IN THIS PAPER AND ITS POTENTIAL SIGNIFICANCE TO THE FIELD

In this manuscript, authors present an RNAi approach for the marine polychaete Streblospio benedicti, a species used for developmental evolution and evolutionary genetics studies as it has two different larval forms in a single species. The authors report two different methods for dsRNA delivery, one by feed bacteria expressing dsRNA to early larvae and another method by microinject in vitro transcribed dsRNA in juvenile worms. They report both a successful and an unsuccessful case, indicating that RNAi efficiency likely depends on the target gene, developmental timing, or dsRNA dosage. Overall, this is an informative contribution that will help people who study other similar species, or life stages where temporal control of gene knock down is needed. The methods presented are written in detail and are appropriate. Parallel assessments such as in situ hybridizations and qPCRs were used. However, the manuscript will benefit from some added details, figure and text clarifications.

SUGGESTIONS TO AUTHORS

Major points
Please insert line numbering and page numbers into your pdf to make it easier for reviewers to refer to specific lines and pages in the manuscript. It takes significantly more time to review a paper without this easy addition.

Section: Results 2.3
- The authors mention quantification in the first sentence, but I don't see that for HCRs, do they mean something else? If they mean Supplement Data 8, this tab in the spreadsheet is really hard to follow. If the authors plot these numbers and integrate them into the HCR figure as quantification, that would justify the verbiage, and will do more justice to this experiment in the main figure.

We are not quantifying the HCR expression. We modified the text in this updated version and added the sentence: We quantified gene expression reduction in differences between our treatments using qPCR and visualized mRNA expression patterns using HCR in situs for larval feeding experiments. L 131-134.

© 2026. Published by The Company of Biologists under the terms of the Creative Commons Attribution License (https://creativecommons.org/licenses/by/4.0/).

- "Although no significant differences in the reduction…" - Sentence missing verb, or it has weird structure that makes it hard to follow.

We modified the sentence and added it to the document. "No significant differences were detected in the expression levels for the F-actin feeding experiment:" P6, L 152-155.

Section 2.4
- "We injected in vitro transcribed dsRNA (120 ng/ µL)"
o Can the authors comment on why these particular concentrations were chosen, and whether they tried other concentrations? This would be helpful for others who want to test similar methods, since this is a methods paper.
o Did they do any initial toxicity tests with control dsRNA for what is the max non-toxic concentration that can be used? Same for larval feeding.

We added to the methods section, the reason that we decided to use 120 ng/ µL. " In a preliminary experiment, we injected two different R-opsin dsRNA concentrations: 120 ng/ µL and 300 ng/ µL in juvenile worms. However, we decided to use the lowest concentration because there was a reduction of the expression levels of the R-opsin gene in samples injected with the lowest concentration after 6 h. We considered that 120 ng/ µL was ideal for our experiment" P10, L-275-279. Even very low levels of dsRNA are effective in some invertebrates such as *C. elegans* due to cellular propagation. We also wanted to avoid any potential toxicity by adding too much dsRNA.

Figure 3 is not doing justice to the results of this paper. There are multiple issues with it:
- Labels are missing
- Number of samples not reported
- Variation in phenotypes unclear because a single image is shown, and the variation is buried into a difficult/unclear supplementary table (suppl. 8)
- Some of the ropsin spots are hard to differentiate. At least digital zooms of these can be provided, not necessarily new photos.
Figure 3 has been modified, and the labels are reported in the updated version. We included some figures of larvae showing the R- opsin domains in the supplementary data.

Results: Sections 2.2 and 2.4
- Please clarify early in the text what constitutes the control for both feeding and injection experiments.
Samples targeting non-dsRNA were fed bacteria from a transformed positive colony with the L4440 plasmid where no gene was inserted (MCS dsRNA) and the non-treatment control samples were wild type early swimming larvae (3 replicates of 25 larvae) (see Methods). P9, L 239-242.
MCS dsRNA (120 ng/ µL) were used as control samples targeting non-dsRNA for side effects of dsRNA and the control samples were injected with Rhodamine (see Methods). P10, L280-2866.

Page 7
- "Therefore, three hours of ingesting dsRNA-expressing bacteria in early planktotrophic larvae was sufficient for the larvae to ingest bacteria and reduce Ropsin gene expression (Figure 2; Supplementary Data 2)." --- The supplementary data only includes F-actin dsRNA, not R-opsin.

The Ct values of the qPCR data of the feeding experiment and the microinjection are included in the second page of the excel sheet (Supplementary Data). Table S2 contains the Ct values of the F-actin dsRNA of the feeding experiment, Table S1 contains the Ct values of the R-opsin dsRNA of the feeding experiment, Table S4 contains the Ct values of the F-actin dsRNA of the microinjection experiment, Table S5 contains the Ct values of the R-opsin dsRNA of the microinjection experiment

Methods
Section 4.2.
- How were larvae processed for RNA extraction? Clarify whether they were pooled and how many were pooled.
The information is in the text: "Clutches of planktotrophic larvae (>300 individuals) from the same brood were divided into treatments and control samples of 25 individuals per well. We used three replicates for each timepoint and treatment. Samples targeting non-dsRNA were fed bacteria from

© 2026. Published by The Company of Biologists under the terms of the Creative Commons Attribution License (https://creativecommons.org/licenses/by/4.0/).

a transformed positive colony with the L4440 plasmid where no gene was inserted (MCS dsRNA) and the non-treatment control samples were wild type early swimming larvae. P9, L237-246. P11, L295.

- "The feeding duration was chosen based…." This sentence seems pretty important but hard to follow and vague for it to be useful to others.
We added a paragraph about the feeding duration and the reason we choose 3 hours: P,9, L 233-236.

In the same sentence "under laboratory conditions": What does this mean? Isn't everything under lab conditions???
We deleted the sentence (under lab conditions) in the updated version of the manuscript.

In the same sentence "after 3h larvae died…" Unclear, were they dying because of concentration? Why did they die after 3 hours and how did this help choosing the duration for feeding? Was being in the presence of E. coli longer than 3h the issue? Is it a hard cut off, if so that seems odd. Please be more clear and specific.

We added a paragraph about the feeding duration and the reason we choose 3 hours: P9, L 233-236.

- MCS: what's the base pair length of this region?
The length of this region is 340 bp. P5, L 124-125.

- 4h: Shouldn't this be 6h?
After feeding planktotrophic larvae for 3 h, the larvae were washed twice with artificial seawater. Gene expression was measured at two timepoints, 4 h and 12 h after washing the larvae samples that were fed.

- In this section, can the authors clarify if they split each batch into two or did they use different replicates for different assays (HCR vs qPCR)
- I think throughout the text and also at the end of this section, it would be good to use more clear language for controls. Here the larvae they mention in the very last sentence is better to call No Treatment Controls (since MCS is a dsRNA control)

We added a new paragraph to the updated document: "Clutches of planktotrophic larvae (>300 individuals) from the same brood were divided into treatments and control samples of 25 individuals per well. We used three replicates for each timepoint and treatment. Samples targeting non-dsRNA were fed bacteria from a transformed positive colony with the L4440 plasmid where no gene was inserted (MCS dsRNA) and the non-treatment control samples were wild type early swimming larvae (3 replicates of 25 larvae). After feeding planktotrophic larvae for 3 h, the larvae were washed twice with artificial seawater. Gene expression was measured at two timepoints, 4 h and 12 h after washing the larvae samples that were fed. Larvae from each treatment were either collected for RNA extraction (for qPCR) or fixed for HCR imaging consisting of three replicates (Figure S6)" P9, L237-246.

Section 4.3
- How many worms were injected per condition? How did you process them for RNA extraction, did you pool individuals?
A single individual worm was considered as one replicate and three replicates for each timepoint were performed. P10, L288-290. They were not pooled.

- "such as early juveniles and swimming lecithotrophic larvae" -- does this mean earlier than 2 weeks? Hard to know for people not familiar with this organism
We added (earlier than two weeks) in the text, P9, L262.

Section 4.4
- It is important to report the primer efficiency for each primer set. If the authors have this, please indicate.
We added the primer efficiency to each set of primers for the qPCR experiment.

- Additionally, please include information about the RNA quality obtained in the experiments.

© 2026. Published by The Company of Biologists under the terms of the Creative Commons Attribution License (https://creativecommons.org/licenses/by/4.0/).

We added the RNA concentration of the larvae of the feeding experiment and the dsRNA microinjections of injected juveniles in Table S7. We did not check the RNA quality as we commonly use this RNA protocol without issue. For different experiments such as library prep, we do use the bioanalizer to check RNA quality and results from this extraction method have been good.

- "Three independent repeats for each replicate were examined and the samples were subjected" -- Can the authors clarify if this means they did qPCRs 3x for same samples? Or whether they had 3 replicates of the same sample per qPCR run?

Yes, we did three qPCRs runs for the same sample (replicate). This is sufficient to show a true difference compared to the control housekeeping gene. P11, L308-311.

Figures
Figure 1
- Please Indicate the number/ percentage of observations supporting the described Phenotype.
Fig 1 is just representative pictures of the expression patterns of R-opsin and F-actin in wild type specimens at different developmental stages. These expression patterns are consistent.

- Please also outline the shape of the larva with a dashed line.
Changes were made to clarify to figures

- Please add titles for each row group (A-E) and (F-J). It is difficult to go back and forth between the legend and images. Example: A-E rows: R-opsin F-J rows: F-actin
Changes were made in the updated figure.

Figure 3
- Similarly, the figure would be clearer if row titles and the number/ percentage of observations supporting phenotypes were included (see comment on Figure 1). labels for control vs ropsin dsRNA etc. 4 vs 12 h.
We added labels to each panel of Figure 3. We added in the figure legend “For the total number of observations supporting phenotypes for each treatment and timepoint (see Table S3)” 538-540. In the methods section, it is indicated the methodology that we used to count the phenotypes for each treatment and timepoint “For the total number of observations supporting phenotype for each treatment and timepoint (see Table S3)” Lines 339-341.

- It would be really helpful to show some more examples of phenotypes in the supplementary. When the larvae were missing eyes, how were they categorized in terms of the phenotype? If one eye was missing vs 2 vs 3? supple 8 data is confusing, as there is not much explanation for it. It will be better to integrate those quantifications here, and also show some different phenotype examples in a supplementary figure
We added additional drawings to the main text and figures to the supplementary material to show the location of eyespots. In Table S3 (control vs non-control) larvae have three eyes. Unfortunately, larvae lose their eye pigmentation after fixation, so we did not include DIC images.

Figures 2 and 4:
- Please specify the number of samples analyzed. Did you have three replicates per sample, or three biological replicates in total? If only three biological replicates were used, it would be advisable to increase the number of experiments if possible. The limited sample size may explain why no differences were observed for F-actin.

Three independent repeats (3X qpCR) for each of the replicates (3 replicates) were examined and the samples were subjected to 40 cycles of amplification in a QuantStudio 6 Pro Real-Time PCR System P11, L308-311.

Figure Supplement 3
- Please, mention the number or percentage of observations supporting phenotype.
These pictures are an illustration of how juveniles were injected in the lumen and gut with dsRNA + Rhodamine. It is not showing a phenotype.

© 2026. Published by The Company of Biologists under the terms of the Creative Commons Attribution License (https://creativecommons.org/licenses/by/4.0/).

Minor points
Section: Results 2.1
It would be clearer to organize the text following the developmental sequence (as shown in the figure): from the trochophore to the juvenile stage.
Changes were made and the description of the expression patterns follow a consecutive order from the trochophore to the early juvenile stage. P2,3 L, 85-106. We show this in the drawings in fig 1 as well.

Figure 2: Consider spelling out what MCS is in figure legend
We added the meaning of MCS (Multiple cloning site of the L4440 plasmid) in Figure 2 and Figure 4.

Figure 4: Figure itself is missing A and B labels
We added the labels A and B in Figure 4.

Figure legends of Suppl Data text: "five o six R-opsin" typo
We corrected the typo mistake in the supplementary data

Second decision letter

MS ID#: dev.205644R1

MS TITLE: A methodology for RNAi gene knockdown in the marine annelid Streblospio benedicti by delivering dsRNA

AUTHORS: Jose Maria Aguilar-Camacho and Christina Zakas

Dear Dr Zakas,

I am happy to tell you that your manuscript has been accepted for publication in Development, pending our standard publication integrity checks.

Reviewer 1

SUMMARY OF THE ADVANCE MADE IN THIS PAPER AND ITS POTENTIAL SIGNIFICANCE TO THE FIELD

As previously stated, this manuscript develops RNAi for mRNA knockdown in the marine annelid Streblospio benedicti, which has two developmental morphs --- a planktotrophic morph and a lecithotrophic morph. They used two separate methods for delivering double-stranded RNA; they fed bacteria expressing the dsRNA to planktotrophic larvae and injected dsRNA into juveniles. The authors attempted knockdown of two genes, R-opsin and F-actin, followed by quantification of mRNA levels by qPCR (larvae and juveniles) and HCR ISH (larvae). Studying development in this annelid has the potential to answer many interesting questions concerning life history evolution and the genetic control of larval versus adult features. The lab has already generated multiple resources for this animal including a culturing system and a high-quality, chromosome-level assembly. I think this manuscript is an important step forward in being able to test gene function in S. benedicti, and publication would benefit researchers who want to study this annelid as well as researchers who want to manipulate gene function in other understudied marine larvae. Currently, this manuscript fits as a Techniques and Resources paper since it describes approaches to use RNAi in for planktotrophic larval and juvenile stages of an annelid.

SUGGESTIONS TO AUTHORS

The manuscript has been edited for clarity and readability, and my questions have been generally addressed. The edits to the manuscript make it easier to understand, and the additional methodological details are helpful.

© 2026. Published by The Company of Biologists under the terms of the Creative Commons Attribution License (https://creativecommons.org/licenses/by/4.0/).

I initially asked for more assessment of the larval phenotypes after RNAi. However, based on the authors' responses, the only measurable phenotype is reduction in the number of R-opsin domains. My preference would have been measurement of additional markers to see a change in cell fate or something else besides R-opsin expression; however, it does not seem like this is currently possible because these cells are not characterized in detail.

Reviewer 2

I would like to thank the authors for their comprehensive and thoughtful responses to my previous comments. The revision has greatly improved the clarity and rigour of the manuscript.

1. With regard to the central claim, I am grateful that the authors have updated the title and toned down the generalisation throughout the text, clearly stating that this is a stage-specific delivery system whose efficiency varies depending on the target gene. This properly frames the paper as a Techniques and Resources report.

2. Regarding the specificity controls, although a second non-overlapping dsRNA fragment was not tested, the authors provided the requested BLAST alignment against the genome resource. This confirmed high specificity for R-opsin, though six potential off-target hits for F-actin were also revealed. I am grateful that the authors have explicitly added recommendations in the text to help future researchers optimise sequences independently based on these findings.

3. The biological justification for the R-opsin phenotype (i.e. that it does not alter gross eye morphology, making HCR signal reduction the primary phenotypic validation) is valid, as is the benchmarking against other recent marine invertebrate RNAi papers.

Overall, the authors have addressed my major concerns satisfactorily through textual clarifications and bioinformatic validations. I support the publication of this manuscript.

Reviewer 3

Authors have addressed comments sufficiently or provided explanations where not applicable or feasible.

© 2026. Published by The Company of Biologists under the terms of the Creative Commons Attribution License (https://creativecommons.org/licenses/by/4.0/).
